# Characteristics of wetting behavior under unloading in natural granite residual soil

**DOI:** 10.1038/s41598-025-91749-8

**Published:** 2025-02-28

**Authors:** Song Yin, Jianing Huang, Xinming Li, Yuzhou Sun, Yuru Li, Xianwei Zhang

**Affiliations:** 1 School of Civil Engineering and Architecture, Zhongyuan University of Technology, Zhengzhou, 450007 Henan China; 2 State Key Laboratory of Geomechanics and Geotechnical Engineering, Institute of Rock and Soil Mechanics, Chinese Academy of Sciences, Wuhan, 430071 Hubei China

**Keywords:** Granite residual soil, Unloading, *K*_0_-consolidation, Wetting behavior, Model, Civil engineering, Hydrology, Engineering

## Abstract

Excavation of foundation pits induces stress release in the soil, leading to deformation driven by the redistribution of internal stresses and particle adjustment. Rainfall infiltration further increases soil water content, weakening particle bonding through the dissolution of cementing agents, and inducing additional wetting deformation. However, there has only been limited experimental research examining the deformation behavior of soil under the coupled effects of unloading and wetting, especially in water-rich excavation conditions, where these factors interact dynamically. This study systematically investigates the coupled effects of unloading and wetting on the deformation behavior of natural granite residual soil (GRS) through triaxial tests. The results reveal that the interaction between unloading and wetting amplifies soil deformation, with significant non-linear dependencies on confining pressure and saturation levels. The stress–strain curves of natural GRS under unloading path exhibit strain-hardening behavior, and the vertical wetting deformation decreases with increasing saturation. Furthermore, the study identifies pronounced anisotropic wetting deformation, with tensile wetting deformation significantly exceeding compressive wetting deformation under equivalent stress states. This anisotropy diminishes with increasing confining pressure, highlighting the stress-dependency of wetting deformation behavior. The hyperbolic model shows a larger wetting deformation than the linear model, underscoring its practical significance in designing safer excavation strategies under coupled unloading and wetting conditions. These findings provide a foundation for improving deformation prediction and risk management in geotechnical engineering.

## Introduction

Rainfall infiltration leads to a decrease in matric suction and the softening of the cementing materials between particles in unsaturated soil^1–3^. Under the action of loads, relative sliding, fragmentation, and rearrangement of soil particles can then occur. Therefore, the soil will undergo certain deformations to reach a new equilibrium state; this type of deformation is known as wetting deformation^4^. As a method to study wetting deformation of soil, wetting tests were initially used mainly in research examining the deformation of expansive soil and loess, it was found that fine-grained materials also experience strength loss similar to that of coarse-grained materials after being immersed in water, and this can cause wetting deformation. With further in-depth research on wetting tests, the wetting behavior of coarse-grained earth-rock dams^5^ has also been observed. Extensive research has shown that the wetting-deformation of coarse-grained soil can cause structural damage of the dam, such as settlement, lateral displacement, stress redistribution, and even cracks^6,7^.

The research object of soil wetting has expanded from expansive soil^8–10^ and loess^11–13^ to coarse-grained soil^13–15^, earth-rock dam^16^, and weathered rock and soil^17,18^. The study of the wetting deformation mechanisms of various geotechnical materials has become more thorough, and the corresponding theoretical research has advanced significantly. It can be seen that a large amount of research has been conducted on soil wetting test methods, wetting mechanisms, microstructures, and wetting-deformation theory, and it is believed that the wetting deformation of soil cannot be ignored and is an important factor in controlling the deformation and stability of foundation pits and related buildings and structures^19,20^.

**Fig. 1:**
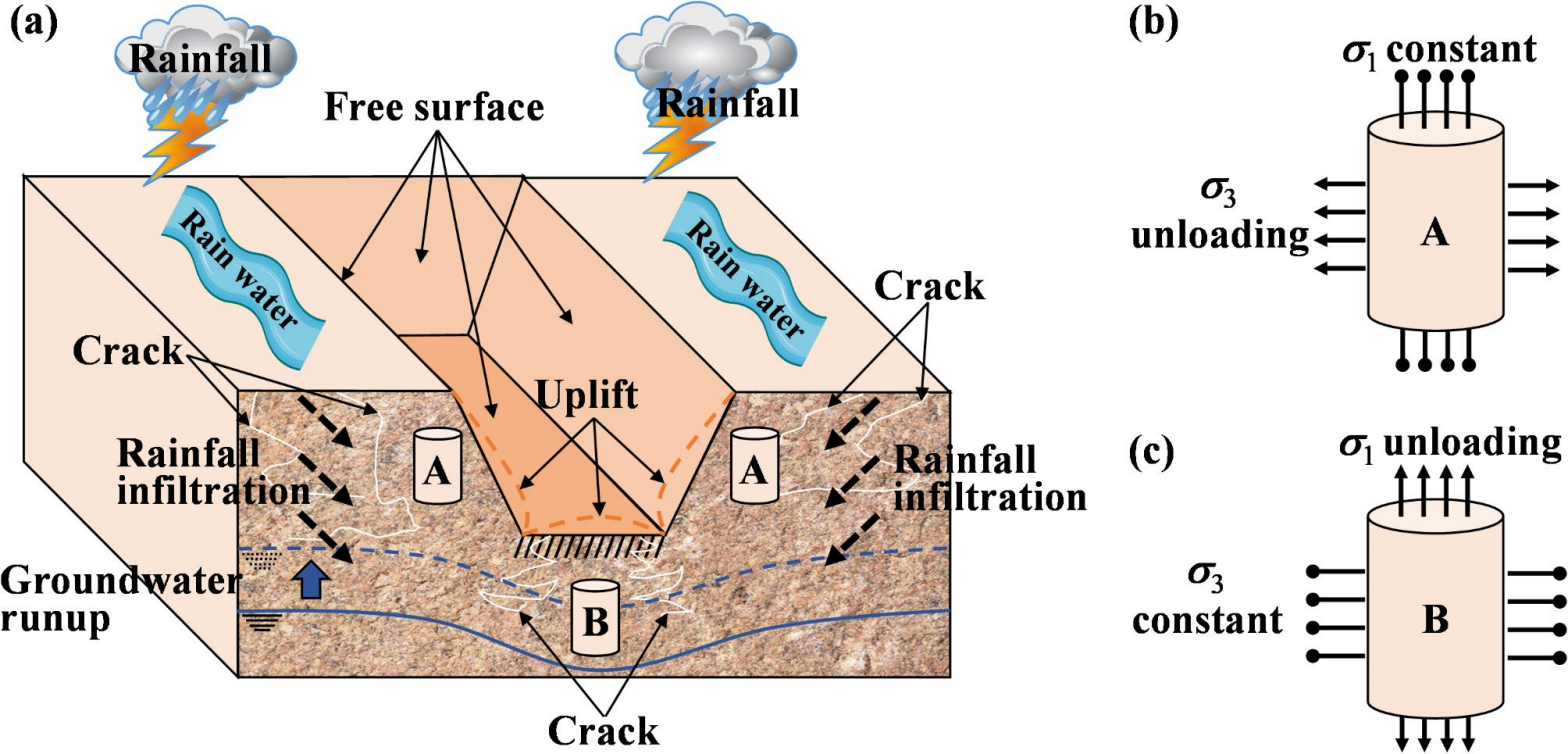
Diagrams illustrating (**a**) deformation of the soil due to rainfall infiltration during the excavation of a foundation pit; (**b**) stress changes of soil unit in area A; (**c**) stress changes of soil unit in area B.

However, conventional stress paths in wetting deformation tests often simulate loading conditions^21^, which do not accurately reflect the unloading effects observed during foundation pit excavation. The redistribution of soil stress in the process of excavating foundation pits leads to a new stress state. Through triaxial unloading stress-path tests and numerical calculations, scholars have confirmed that stress paths have a significant influence on the stability and deformation of foundation pits^22^, and obtaining soil parameters using conventional compression paths is unfavorable to engineering safety. Therefore, the unloading stress path (as shown in Fig. 1) of soil-moisture deformation in foundation-pit excavation in detail, as it is different from the loading stress paths of conventional moisture tests. The interaction between unloading-induced stress redistribution and wetting-induced softening and deformation is critical in foundation-pit engineering, as these coupled effects govern the stability and mechanical response of the soil. To date, there has been limited understanding of this coupling mechanism.

Granite residual soil (GRS) is widely distributed in southeast coastal areas of China, and it is commonly involved in local geotechnical engineering construction. The formation process of GRS is characterized by aluminization, iron enrichment, and salt leaching^23,24^. As a result, the structural cracks and secondary joints remaining after weathering of bedrock evolve into numerous microcracks, forming potential structural failure planes in the soil mass. This in turn has a significant impact on the strength, deformation, and permeability of the soil mass. GRS exhibits strong anisotropy^25^ and is greatly affected by the stress path. Particularly when there is a free surface in excavation, the cracks in soil develop rapidly due to stress concentration, and stress-path effects cannot be ignored. This highlights the necessity of simulating excavation scenarios using unloading stress paths.

The southeastern coastal areas of China are facing decreasing availability of land for construction. To accommodate urban expansion, there has been an increasing number of deep-foundation pit projects for underground infrastructure, such as subway systems, and for high-rise buildings. Due to the inherent characteristics of GRS, the abundant rainfall in the climate of southeastern coastal China, and the insufficient understanding of the unique properties of GRS by engineering designers and construction personnel, inappropriate technical measures are often adopted, leading to frequent geotechnical engineering disasters associated with GRS-related excavation projects^26^. Studies have shown that soil is usually in a state of *K*_0_-consolidation stress under natural conditions^27^, and the initial deviator stress often has a significant influence on the wetting deformation characteristics of soil under an unloading stress path. Therefore, considering the characteristics of actual engineering excavation, it is necessary to carry out targeted research on the effect of wetting of natural GRS under the coupled conditions of unloading and rainfall infiltration under *K*_0_-consolidation stress.

To date, researchers^28–30^ have conducted extensive research on computational models of soil wetting deformation. However, the models developed by these researchers are all focused on the wetting deformation of soils under loading paths; it remains unreported whether these models are applicable for predicting the wetting deformation of soils under unloading paths.

For these reasons, in this work, we used a GDS Instruments stress-path triaxial testing system to conduct wetting tests on GRS under different unloading stress paths and shearing states in the *K*_0_-consolidation state. The aim was to study the mechanical response characteristics of GRS during the excavation of a foundation pit under rainfall infiltration conditions and evaluate the applicability of the wetting model under unloading stress paths based on the test results. The study of wetting deformation in soils provides critical insights for engineering practices, especially in foundation pit excavations where rainfall infiltration or groundwater exposure can induce significant deformation. Accurate prediction and effective control of wetting deformation are vital for ensuring the stability of foundation pits and minimizing associated risks. This research provides experimental data and predictive models that can guide the design of support structures and the implementation of drainage measures to mitigate wetting-induced deformation.

**Fig. 2:**
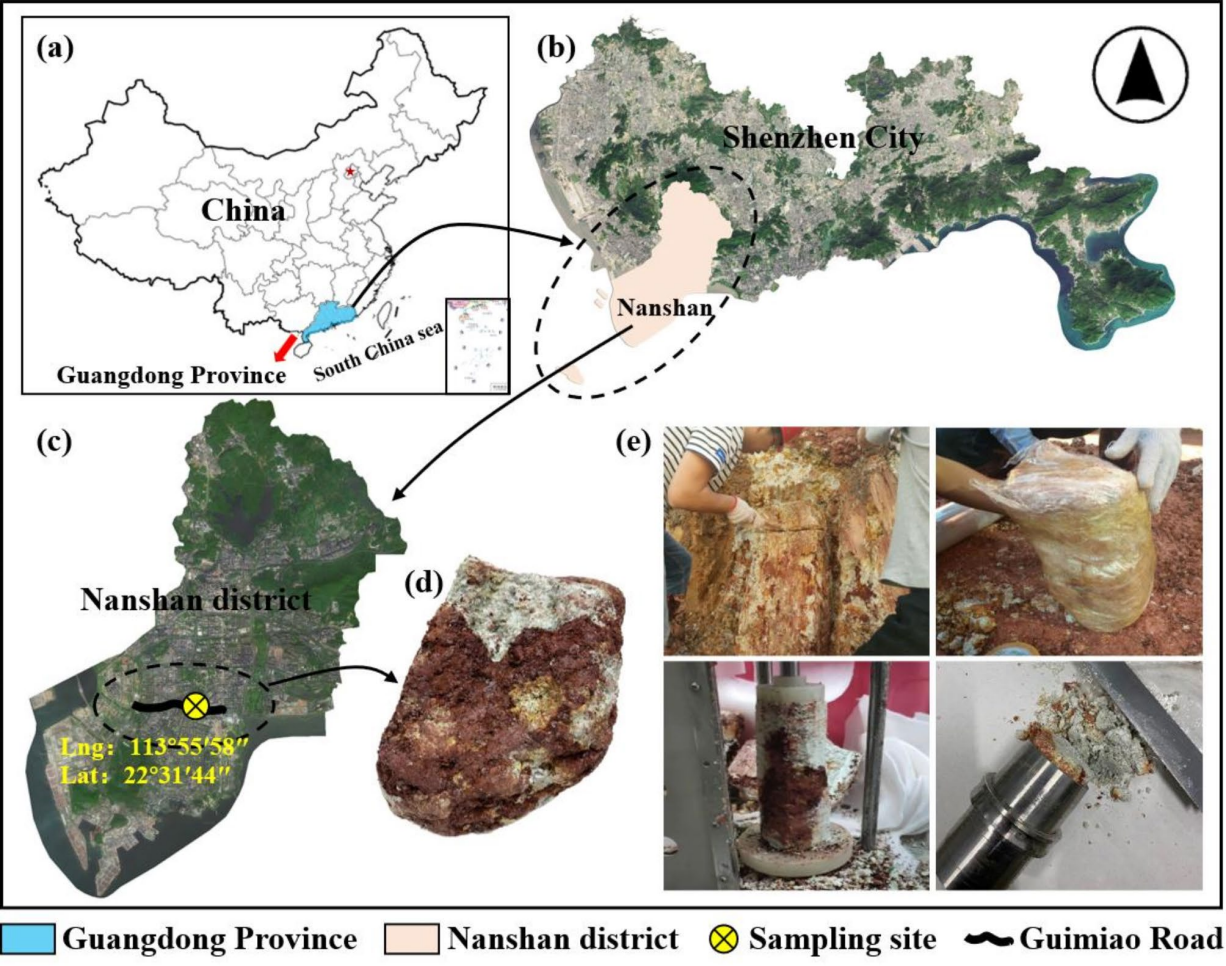
Location of sampling site and sampling process: (**a**) Guangdong, China; (**b**) Shenzhen, Guangdong; (**c**) Nanshan District, Shenzhen; (**d**) The appearance of a sample; (**e**) Sampling and sample preparation. The maps were created with ArcGIS Online (https://www.arcgis.com/apps/mapviewer/index.html) and Microsoft PowerPoint (Version: 2411, https://www.microsoft.com).

## Materials and methodology

### Characterization of GRS

**Fig. 3:**
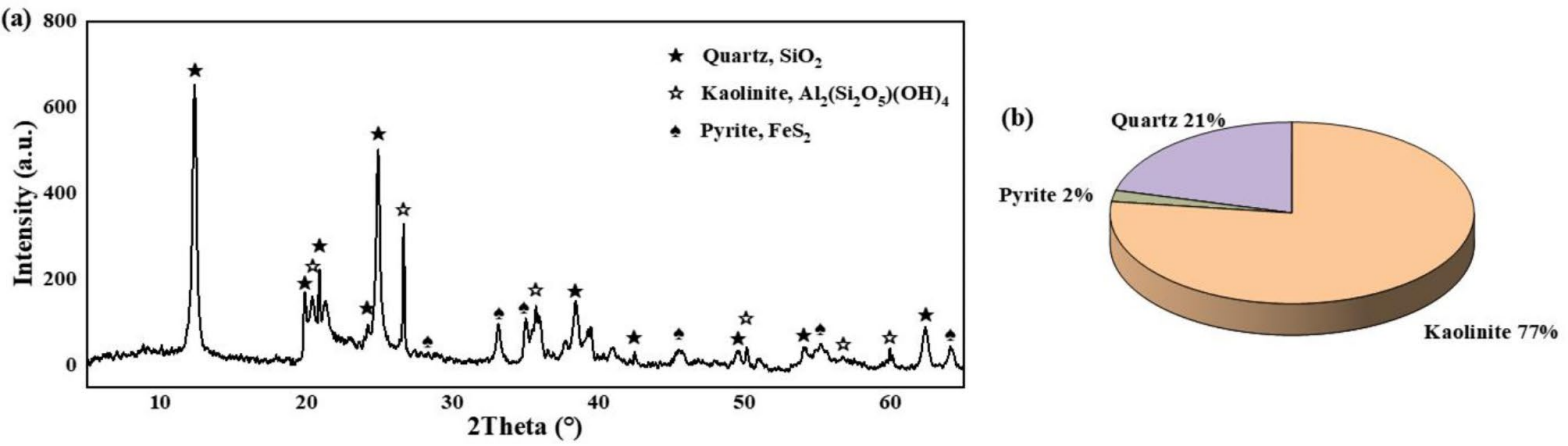
XRD test results: (**a**) spectrum; (**b**) mineral composition.

**Fig. 4:**
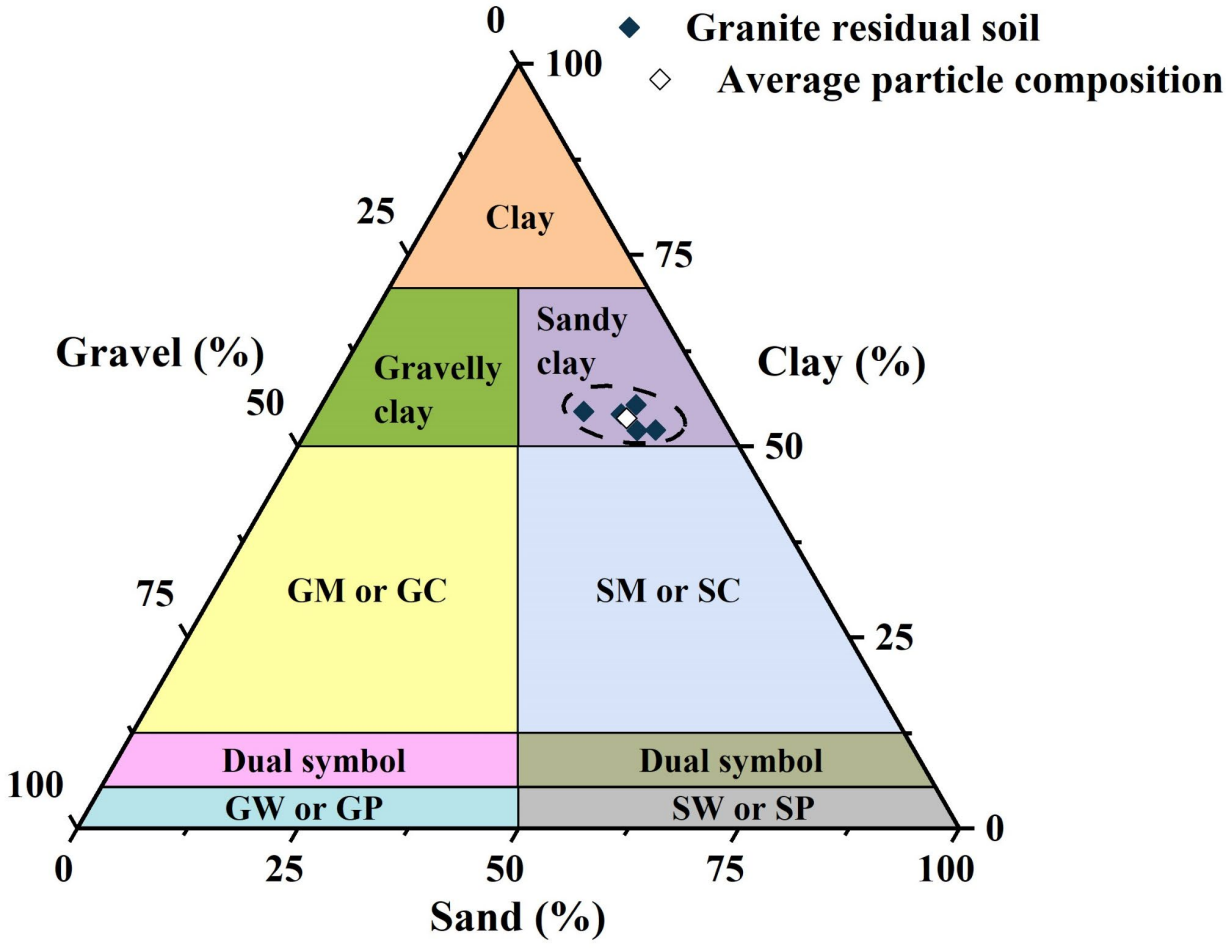
Particle composition of GRS according to the Unified Soil Classification System.

The soil samplings were conducted in Shenzhen City, Guangdong Province, China^31^, as shown in Fig. 2. The soil samples were collected using a manual sampling method at a depth of approximately 4 m, and the undisturbed soil samples were cut into cubes (30 cm × 30 cm × 30 cm). The block-like undisturbed GRS samples were wrapped with geotechnical preservation film and double-wrapped with plastic shock-absorbing membranes to reduce disturbance during transportation. Table 1 presents the physical characteristic of the GRS. In the x-ray diffraction (XRD) analysis (see Fig. 3), it was found that the content of hematite in the GRS was 9.29%. Previous studies have shown that hematite, as a cementing agent, can improve the strength of residual soil. Furthermore, the cemented structure was also leads to be linked to the strength anisotropy of GRS^32^. The results of XRD analysis showed that the GRS was mainly composed of the clay mineral kaolinite, with a content of about 77%. The soil was also found to contain quartz, pyrite, and other non-clay minerals. Most of the minerals, such as feldspar and biotite, had weathered to kaolinite, indicating a high degree of weathering of Shenzhen GRS. As shown in the particle composition in Fig. 4, the selected soil was classified as sandy clay according to the ASTM^33^. Such a wide range of particle composition in residual soil is rare, reflecting the role of weathering conditions in the formation of GRS.

**Table 1:** Average values of physical properties and mineral composition of the GRS samples.

Initial saturation (%)	Natural density (g/cm^3^)		Natural moisture content (%)		Void ratio	Specific gravity	Liquid limit (%)	Plastic limit (%)	Plasticity index
94.40	1.89		30.54		0.88	2.72	48.80	31.00	17.80

Average particle composition (%)						Mineral composition (%)			
Gravel		Sand		Clay		SiO_2_	Al_2_O_3_	Fe_2_O_3_	Others
10.76		36.25		52.99		52.56	34.53	9.29	3.62

### Test methodology

**Fig. 5:**
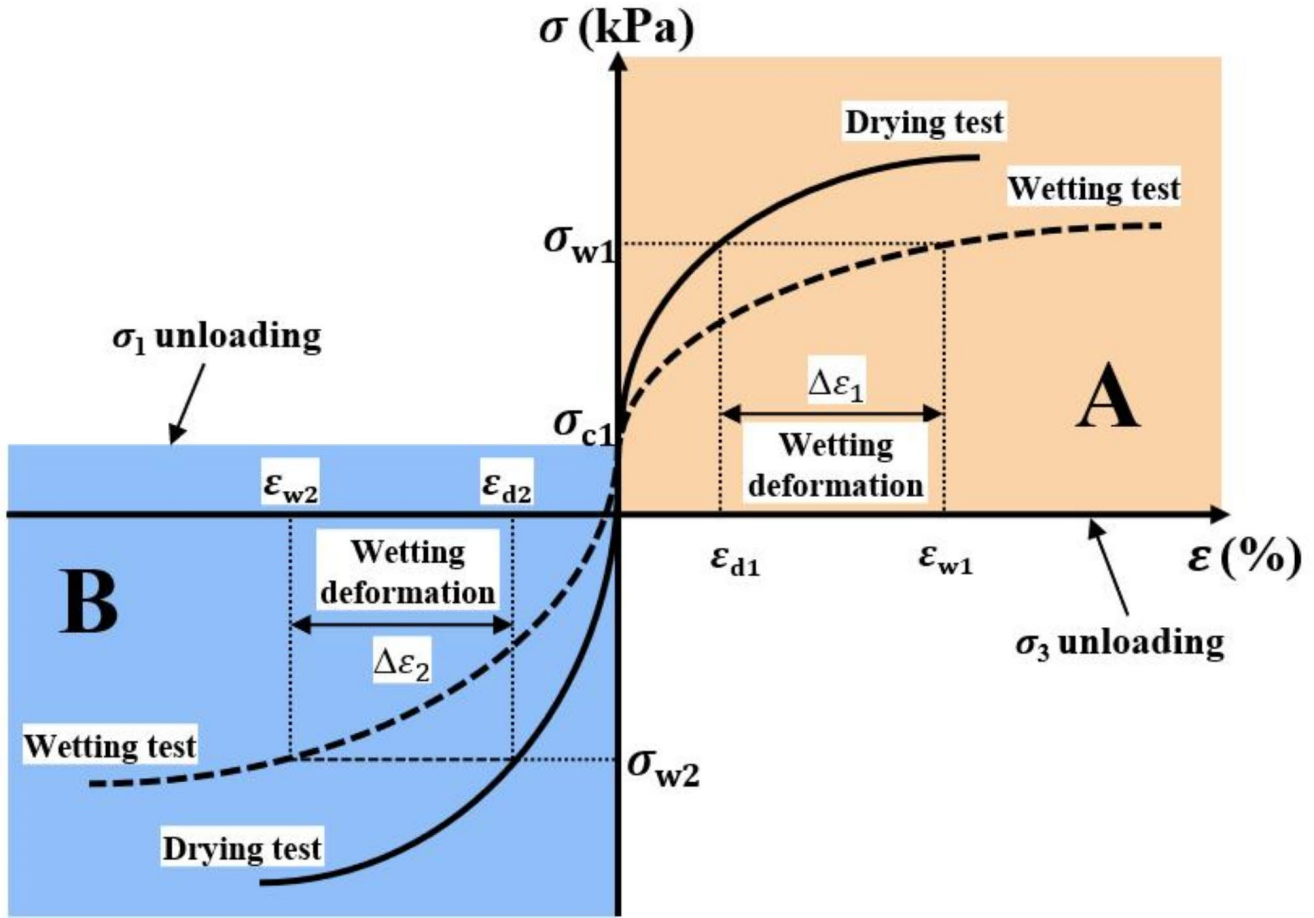
Stress–strain diagram of double-line wetting tests.

This study employed the double-line method to conduct wetting tests on GRS, using two different unloading stress paths to simulate the unloading processes of soil at different locations during excavation. The non-excavated soil on both sides of the foundation pit (region A in Fig. 1a) experiences a reduction in lateral pressure due to lateral excavation (Fig. 1b), which can be simulated in triaxial test by reducing the confining pressure (stress path shown in region A of Fig. 5). The soil at the bottom of the foundation pit (region B in Fig. 1a) experiences a reduction in overburden pressure due to the excavation of the upper soil (Fig. 1c), which can be simulated in triaxial test by reducing the axial pressure (stress path shown in region B of Fig. 5). These two unloading paths consisted of a reduction in lateral stress with constant axial stress, and a reduction in axial stress with constant lateral stress, respectively, as shown in Fig. 5. The three saturation levels used in this study were chosen based on field conditions observed at the sampling site. During hot weather, the lowest water content of GRS was 13%, corresponding to a saturation level of 40.18%. Drainage measures implemented during foundation pit excavation lowered the groundwater level, reducing the saturation level of previously saturated soils to an average of 80.36%. Under heavy rainfall conditions, the soil became fully saturated, with a saturation level of 100%. So, the tests include both unsaturated samples (with degrees of saturation at 40% and 80%) and saturated samples (with a degree of saturation at 100%) to capture the wetting deformation characteristics of soil during the transition from low saturation to full saturation, induced by rainfall infiltration in the context of excavation processes. This setup allows us to investigate the wetting deformation as the soil transitions from an unsaturated to a saturated state under unloading conditions. After conducting unloading tests on both dry and wet samples, stress–strain curves of specimens with two different degrees of saturation were obtained (drying test and wetting test in Fig. 5). At a certain stress level (such as σ_w1_), the wetting deformation of the soil was equal to the difference in strain under the same stress level between the wet and dry samples (*ε*_w1_ and *ε*_d1_).

**Fig. 6:**
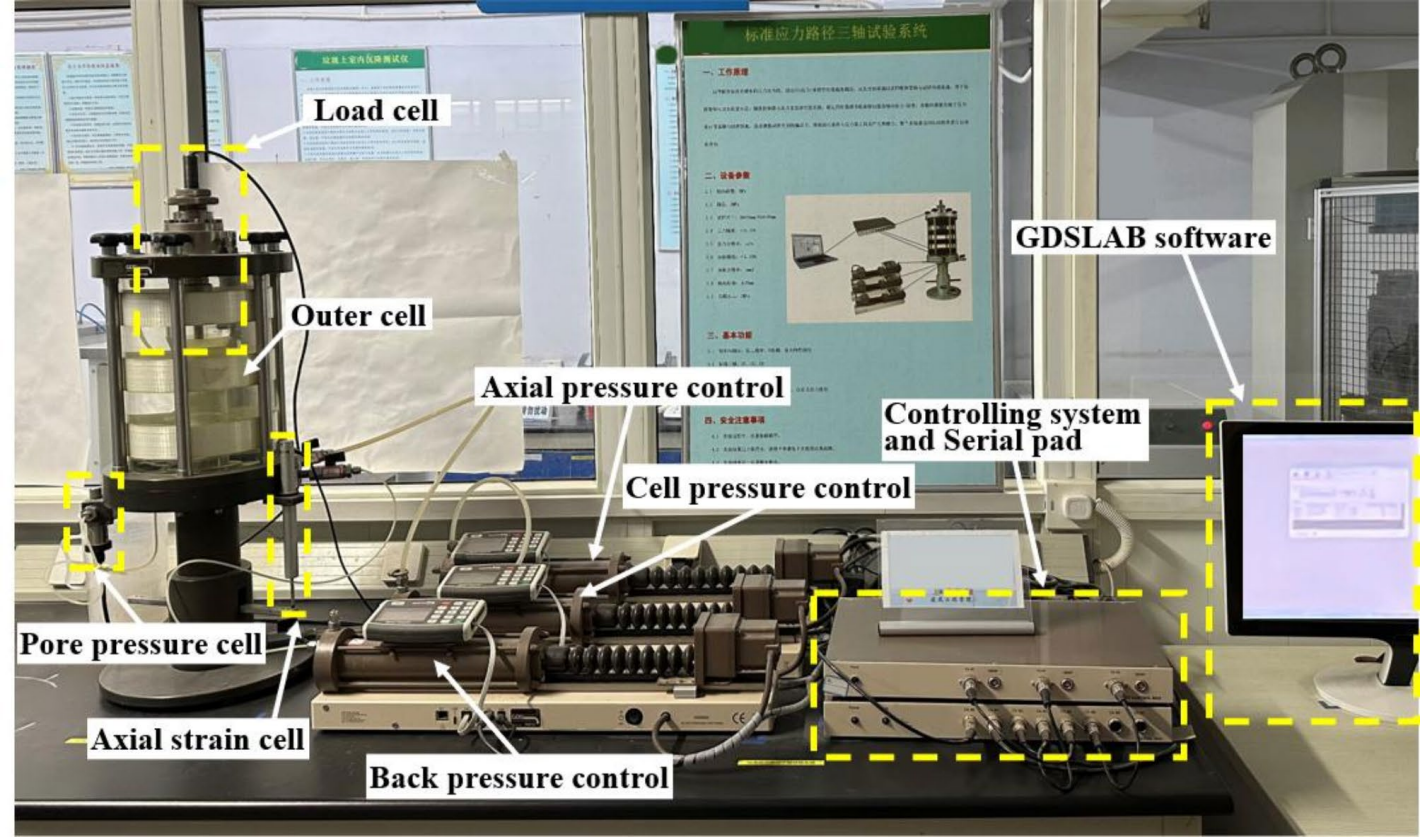
GDS stress path triaxial apparatus.

**Fig. 7:**
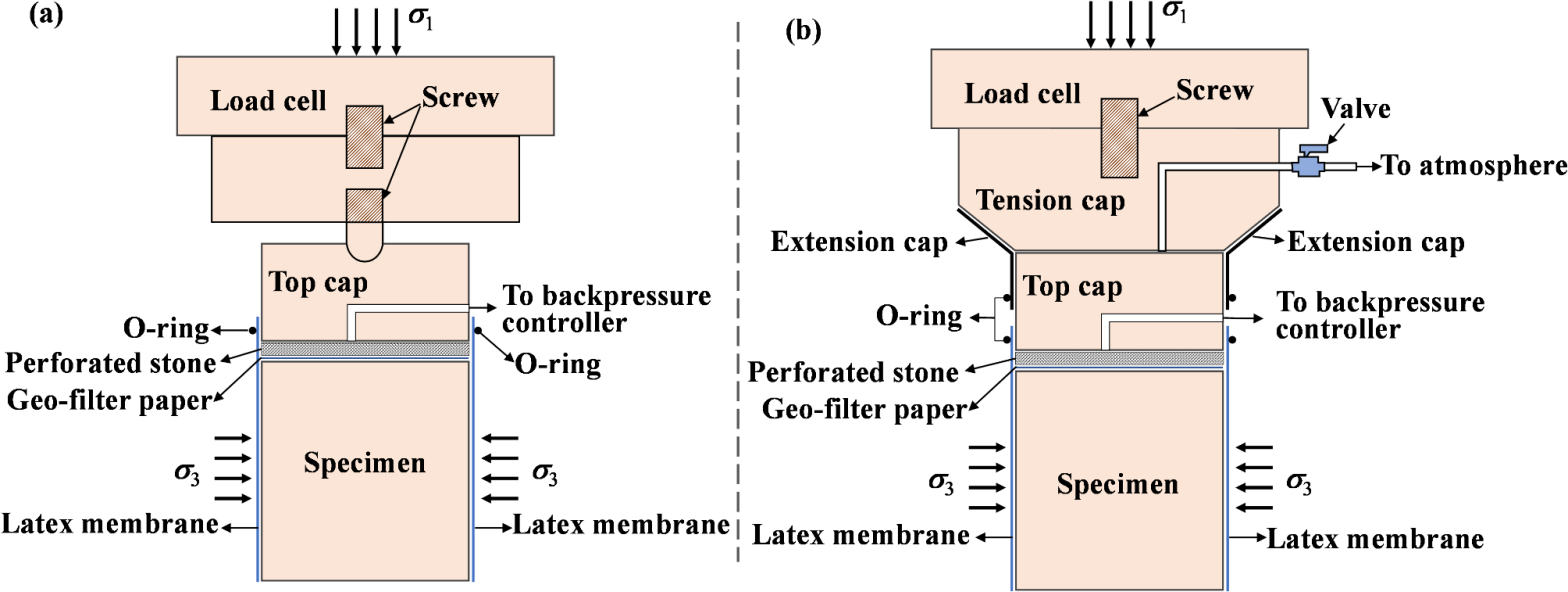
Methodologies of the tests: (**a**) constant σ_1_, unloading σ_3_; (**b**) unloading σ_1_, constant σ_3_.

A GDS Instruments stress-path triaxial apparatus was used in this work, as shown in Fig. 6, and the fundamental measurement technique used was that presented by Zhao et al.^34^. It should be noted that the triaxial instrument accessories used in different unloading path tests were different. In the unloading path tests with constant σ_1_ and unloading σ_3_, the compression tests of the specimens could be completed using conventional triaxial test accessories (see Fig. 7a); however, in the unloading path test with unloading σ_1_ and constant σ_3_, the help of the extension cap was needed to complete the tests (see Fig. 7b).

The test procedure in this study was as follows.

- Specimen preparation. The soil was gently trimmed into cylindrical specimens 100 mm in height and 50 mm in diameter using a trimming frame for triaxial tesFig. (Fig. 2e); see Yin et al.^31^ for a detailed description of the specimen preparation. The initial saturation of the soil was high, and it was dehumidified to obtain dry specimens. The soil samples were placed in box at constant temperature and humidity, and the temperature and humidity were adjusted to obtain samples with saturations of 40% and 80%, the environmental parameters were set according to the extreme temperature (40℃) and relative humidity (84%) of Shenzhen. The quality of the dehumidified specimens was measured periodically. After the dehumidification specimens reaches the predetermined quality, the specimens were wrapped with Geo-preservative film wrap and left for 24 h to evenly distribute water inside the specimens. The samples with 100% saturation were obtained by the vacuum saturation method and the backpressure saturation method. After being saturated for 6 h using the vacuum saturation method, they were saturated again by the backpressure saturation method on the GDS triaxial apparatus. All samples were saturated under a backpressure of 300 kPa, saturation was monitored using the B-value method, where a B-value greater than 0.97 indicates full saturation.
- Specimen-fixing process. Before fixing the specimens, the controllers were filled and drained to remove bubbles from the top cap, the pedestal of the cell base, and the piping. Wet Geo-filter papers and soaked permeable stones were placed on the top and bottom of each soil specimen. Then, the specimens were placed on the pedestal of the cell base and encased in a customized latex membrane (see Fig. 7). A soft brush was used on the latex membrane to gently remove bubbles between the membrane and the specimen, and the top cap was then fixed. The confining jacket of the specimen was set into place by sealing the membrane to the pedestal and top cap above the specimen using O-rings. An extension cap was needed for unloading σ_1_. A layer of silicone grease was applied to the inner wall of the extension cap, paying attention not to apply too much. The small end on the top cap was then fixed in place. Ensuring that the axial load sensor was located at the top position, the tension cap was observed when fixing the outer cell to ensure that it fell inside the extension cap (as shown in Fig. 7b). The extension cap was not required when unloading σ_3_ was performed (see Fig. 7a). The GDSLAB software package was used to zero-load the cell. The plug of the tension cap pipeline was unscrewed, the position of the load cell was adjusted, and a 0.005 kN axial force was applied using GDSLAB to make the tension cap just contact the top cap. The bolt plug of the tension cap pipeline was then tightened. Finally, the outer cell was filled with water.
- Consolidation and unloading stages. The specimen was anisotropically loaded up to the consolidation pressure, and the corresponding σ_1_ value was set (*K*_0_ = σ_3_ / σ_1_, in this work, *K*_0_ = 0.454). After the consolidation of the specimen, keep the drain valve open for unloading test to simulate the soil drainage conditions of the foundation pit. σ_3_ (or σ_1_) unloading was performed at a rate of 0.02 kPa/min to avoid pore water pressure, and the test was completed after the axial strain of the specimen reached 15% (or − 15%). In this work, the axial strain of the specimen has a positive sign in compression and a negative sign in tension. The test plan is as shown in Table 2. These different confining pressures were chosen to simulate the stress conditions at different burial depths of the soil in the foundation pit.

**Table 2:** Unloading-wetting triaxial test plan.

Test group	Unloading path	Consolidation pressure ${\sigma _{3{\text{c}}}}$ (kPa)	Saturation S_*r*_ (%)
I	Constant σ_1_ unloading σ_3_	50	40, 80, 100
II		100	40, 80, 100
III		200	40, 80, 100
IV	Unloading σ_1_ constant σ_3_	50	40, 80, 100
V		100	40, 80, 100
VI		200	40, 80, 100

## Test results and analysis

### Stress–strain curves of soil

**Fig. 8:**
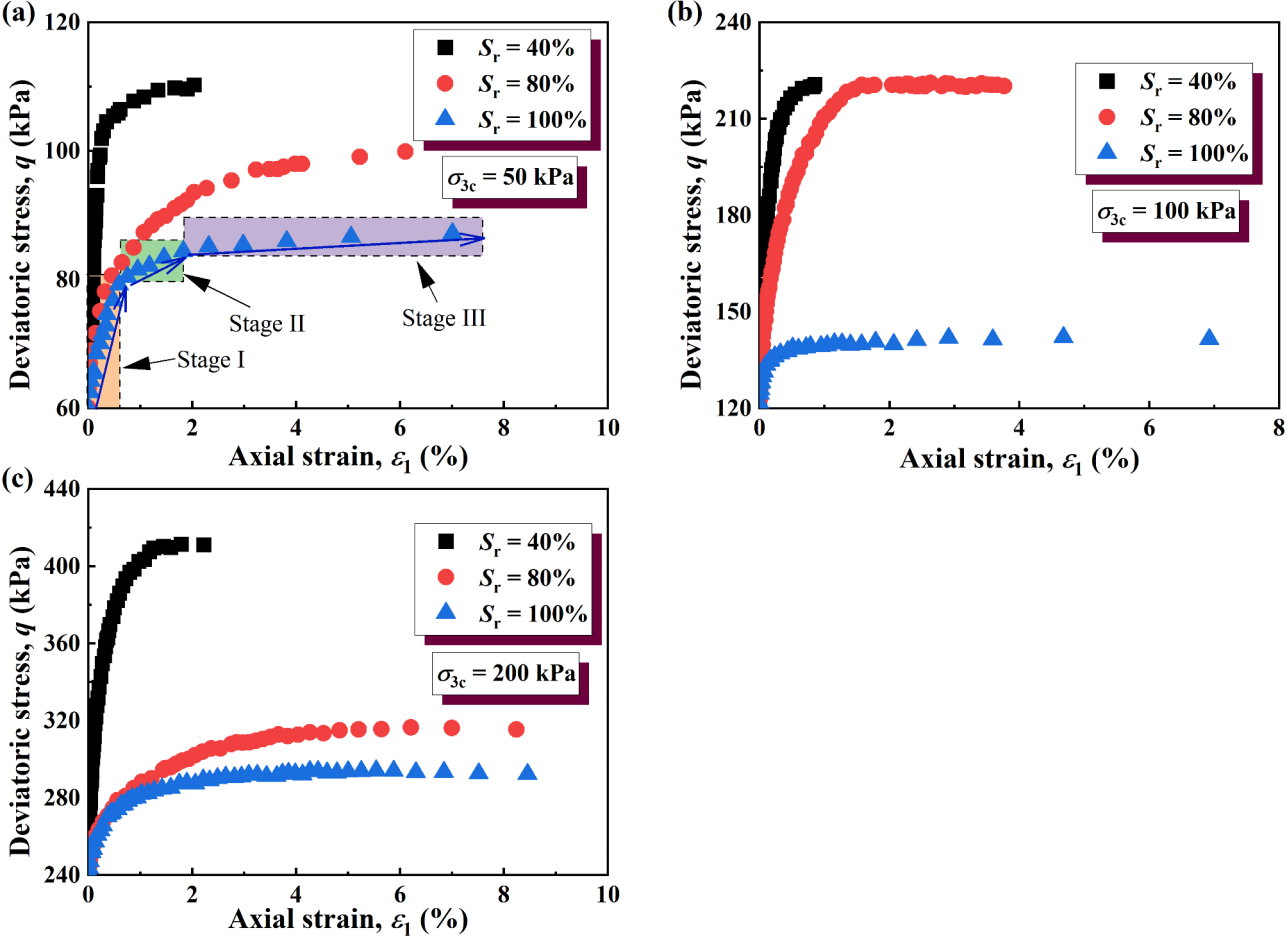
Stress–strain curves of unloading σ_3_: (**a**) σ_3_ = 50 kPa; (**b**) σ_3_ = 100 kPa; (**c**) σ_3_ = 200 kPa.

The stress–strain curves of the unloading σ_3_ test process under σ_3_ = 50, 100, and 200 kPa are shown in Fig. 8. It can be seen that the stress–strain curves of GRS are of the strain-hardening type. In the early stage of strain development (Fig. 8a, Stage I), the deviatoric stress of the soil increases rapidly and approximately linearly. With the continuous development of the strain (Fig. 8a, Stage II), the growth rate of the deviatoric stress borne by the soil decreases. Finally, with the continuous development of the strain (Fig. 8a, Stage III), the deviatoric stress borne by the soil barely increases.

Under the same confining pressure, the lower the saturation, the higher the deviatoric stress of the soils. This may be because the saturated GRS had sufficient water in the pores, and the silt content in the soil specimen was higher. The presence of water reduced matric suction and increased pore water pressure, weakening the effective stress between soil particles. The suction effect in unsaturated soil diminishes as the saturation increases, leading to a significant reduction in effective stress. As the suction decreases, the soil’s ability to resist deformation diminishes, which further contributes to the observed decrease in shear strength. Furthermore, water adsorbed on particle surfaces formed hydration films that enhanced electrostatic repulsion between particles, reducing intergranular bonding. While the GRS with a low degree of saturation exhibited strong cementation due to hematite and other bonding agents, the introduction of water during wetting led to the progressive dissolution of these agents, resulting in weakened cementation and loss of adhesion characteristics, and the softening of iron oxide in water thus also reduced the strength of the soil.

Under the same saturation, the higher the confining pressure, the higher the maximum deviatoric stress of the soils. The reason for this phenomenon may be that initial consolidation confining pressure determines the maximum deviatoric stress increment during unloading. Although this phenomenon was also found in specimens with saturations of 80% and 100%, the increase of the maximum deviatoric stress of the soil with increasing confining pressure was less than that of the specimens with a saturation of 40%. With increasing confining pressure, the increase in the maximum deviatoric stress becomes smaller with increasing water content. According to the shear strength theory for unsaturated soils, this phenomenon can be attributed to the reduction in matric suction as the water content rises, which diminishes the effective stress and thereby weakens the inter-particle bonding and shear strength. Additionally, a possible explanation for this is that water lubricates soil particles by reducing inter-particle friction and weakening particle bonding. At lower water content, thin water films surround the particles, slightly reducing frictional resistance. As water content increases, more free water fills the voids between particles, enhancing the lubrication effect and significantly reducing shear strength by weakening inter-particle interlocking and cementation.

**Fig. 9:**
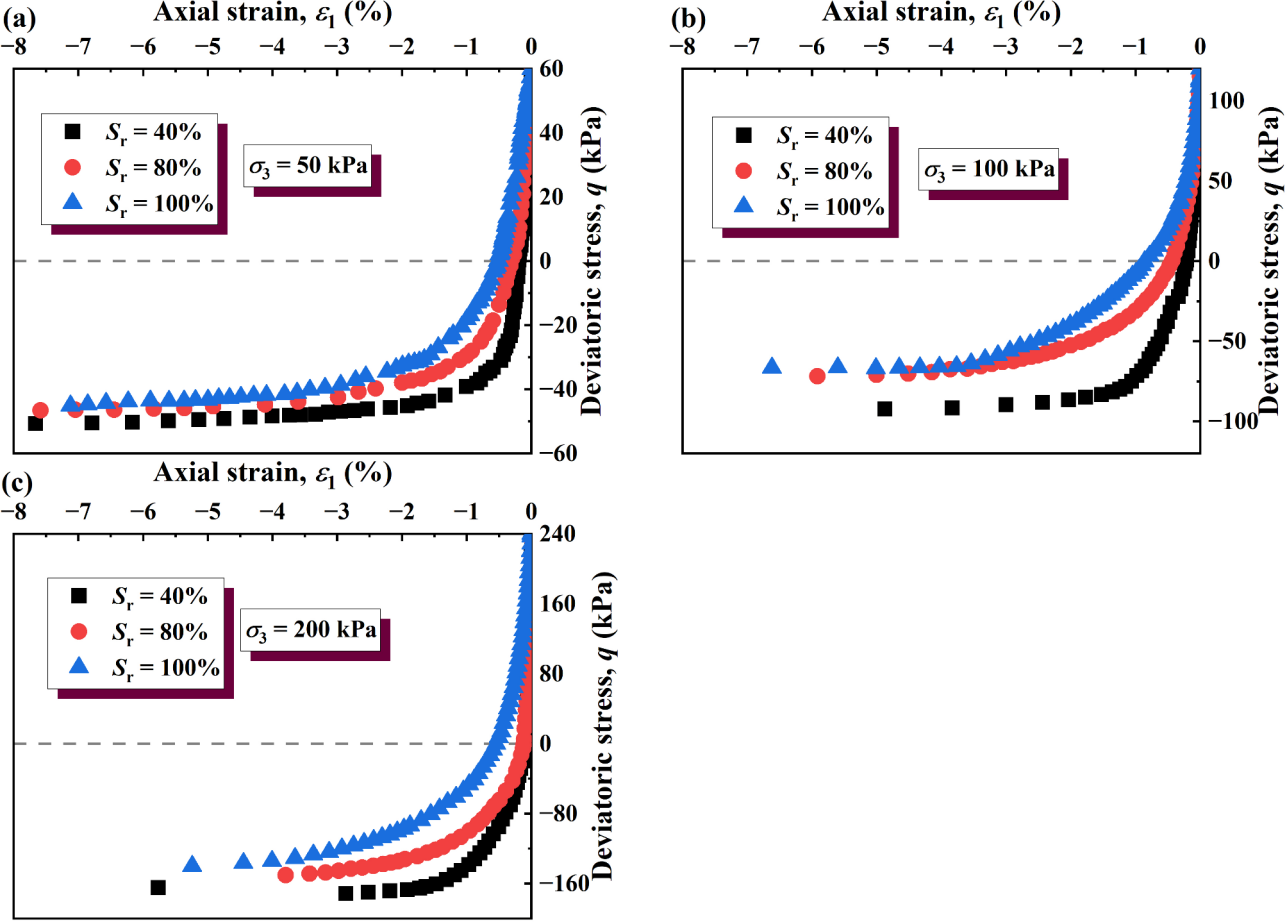
Stress–strain curves of unloading σ_1_: (**a**) σ_3_ = 50 kPa; (**b**) σ_3_ = 100 kPa; (**c**) σ_3_ = 200 kPa.

The stress–strain curves of unloading σ_1_ test processes with σ_3_ = 50, 100, and 200 kPa are shown in Fig. 9. Under the unloading σ_1_, the stress–strain curves were also of the hardening type. The strength of the soil decreases with increasing saturation, which is consistent with the variation of soil strength with saturation under unloading σ_3_. The reason for this decrease in soil strength was consistent with that under unloading σ_3_. It can be speculated that the same mechanism as confining pressure unloading leads to this phenomenon.

The experimental results indicate that the reduction in deviatoric stress due to wetting is significantly amplified under unloading conditions, demonstrating that the combined effect is not merely additive but interactive. The stress-strain curves (Figs. 8 and 9) reveal that under the same confining pressure, the decrease in deviatoric stress with increasing saturation is more pronounced in unloading conditions compared to conventional loading paths. This suggests that the weakening effect of wetting is stress-path dependent, with unloading-induced stress redistribution accelerating the strength loss associated with suction reduction.

This phenomenon can be attributed to the microstructural behavior of GRS, where unloading leads to stress-induced microcracking, which enhances water infiltration. As saturation increases, the loss of matric suction further weakens interparticle bonding, leading to a nonlinear reduction in soil strength.

### Results of wetting deformation

#### Simplified linear model

**Fig. 10:**
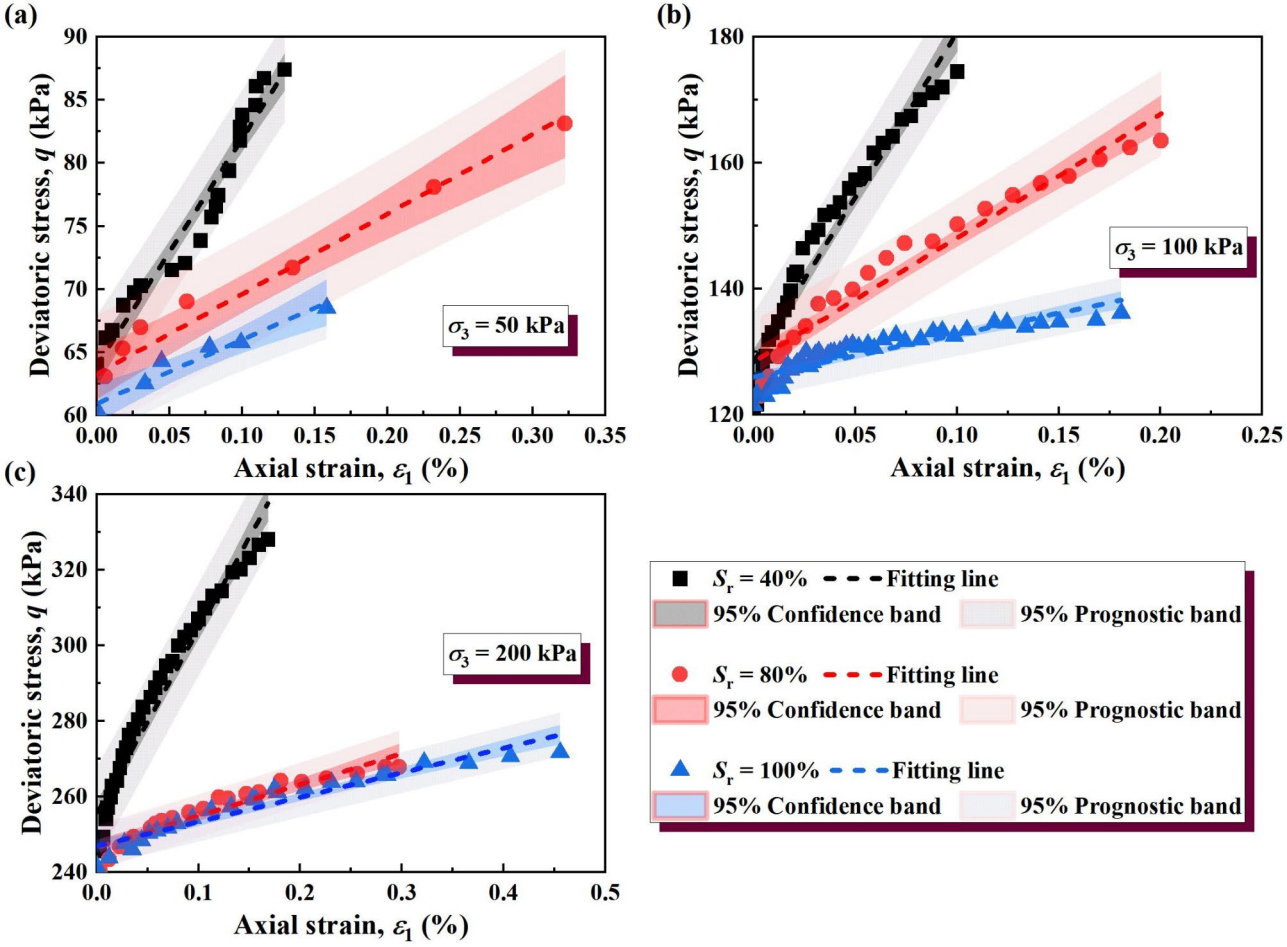
Stress–strain curves of unloading σ_3_ after linear simplification: (**a**) σ_3_ = 50 kPa; (**b**) σ_3_ = 100 kPa; (**c**) σ_3_ = 200 kPa.

**Fig. 11:**
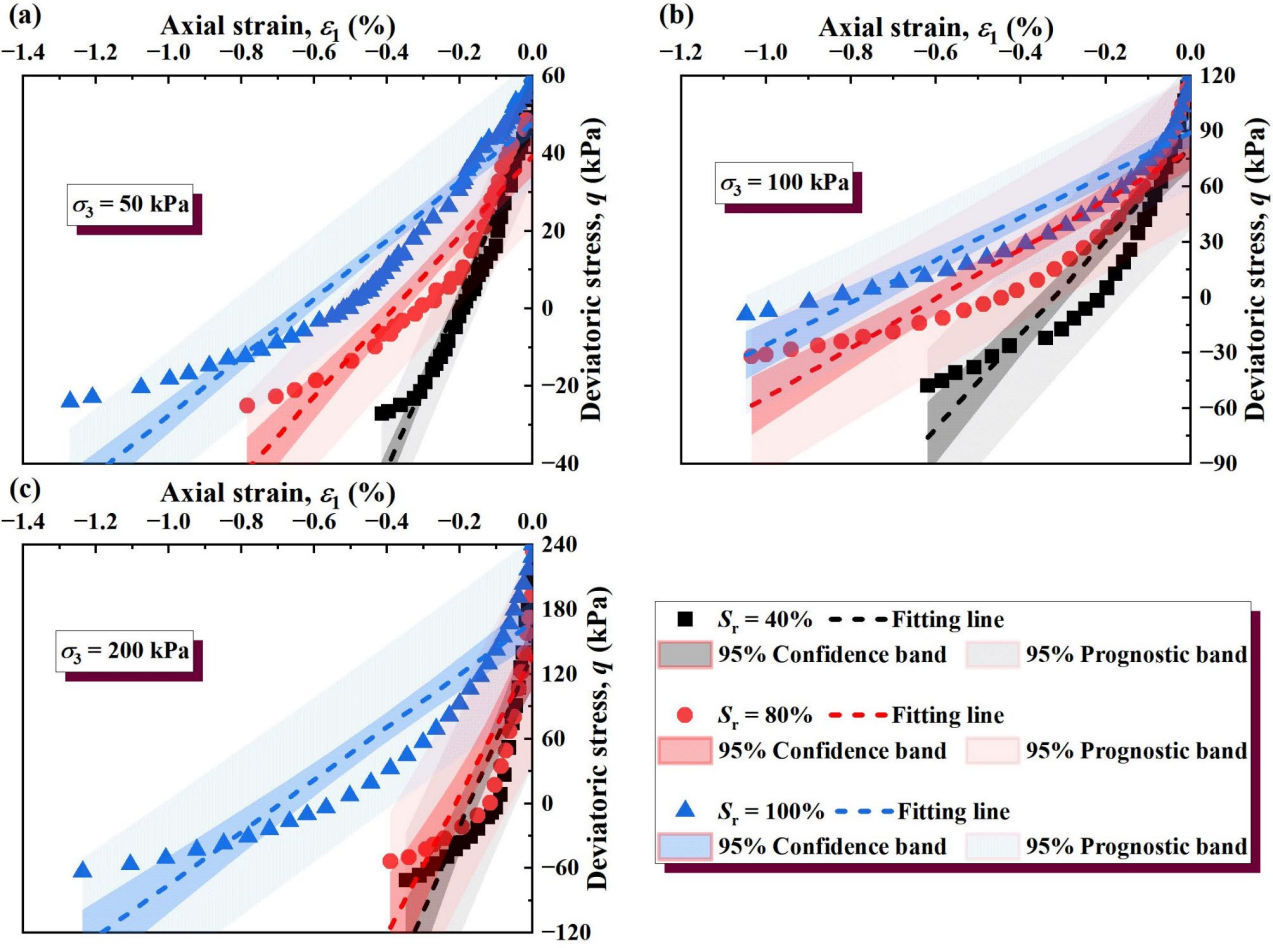
Stress–strain curves of unloading σ_1_ after linear simplification: (**a**) σ_3_ = 50 kPa; (**b**) σ_3_ = 100 kPa; (**c**) σ_3_ = 200 kPa.

**Fig. 12:**
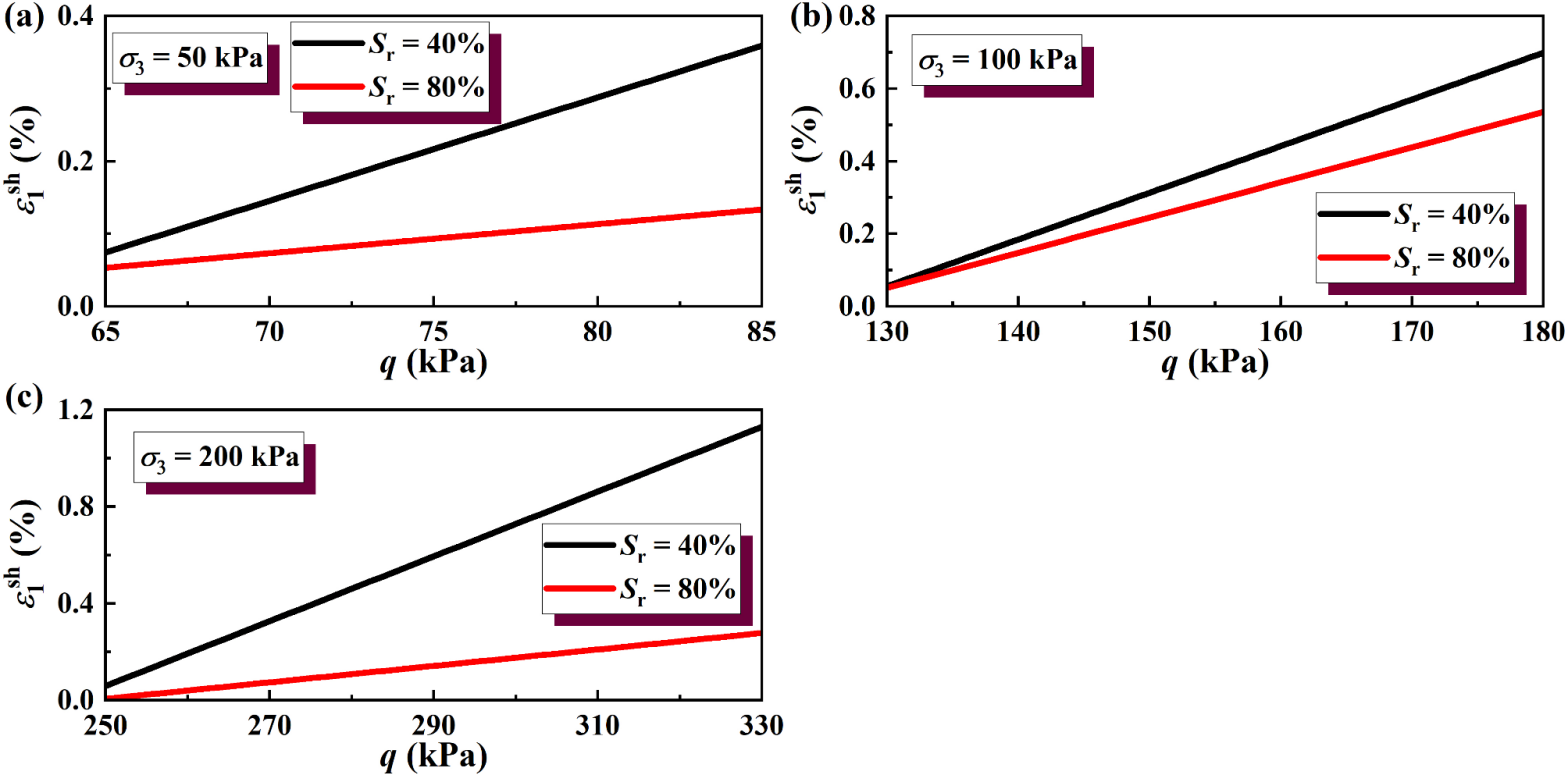
Wetting deformation under unloading σ_3_ calculated by simplified linear model: (**a**) σ_3_ = 50 kPa; (**b**) σ_3_ = 100 kPa; (**c**) σ_3_ = 200 kPa.

**Fig. 13:**
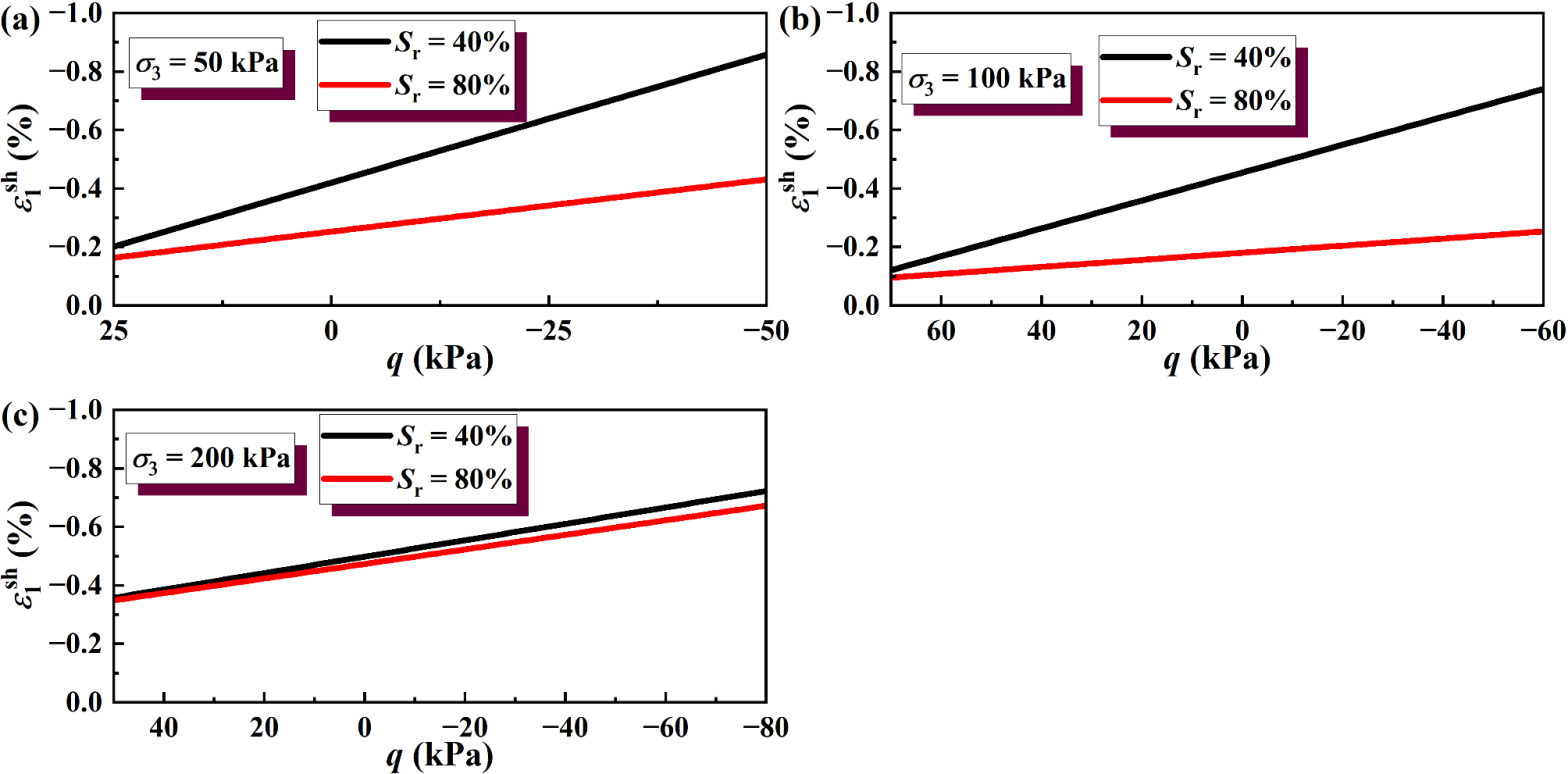
Wetting deformation under unloading σ_1_ calculated by simplified linear model: (**a**) σ_3_ = 50 kPa; (**b**) σ_3_ = 100 kPa; (**c**) σ_3_ = 200 kPa.

The wetting deformation of soil primarily occurs in the initial stage of strain. Therefore, to simplify the analysis of soil stress–strain behavior, we take 80% of the stress–strain curves for fitting. This choice ensures a linear approximation of the dominant deformation region while minimizing the influence of nonlinearity at higher strains, as shown in Figs. 10 and 11. The observed wetting deformation demonstrates elastoplastic behavior, where elastic deformation dominates initially, transitioning into plastic deformation as strain progresses. The simplified linear fitting of the stress–strain relationship can be expressed as

1 $${\varepsilon _1}=\frac{{({\sigma _1} - {\sigma _3}) - t}}{{{E_{\text{t}}}}}$$

where *t* is the intercept of the fitting curve on the *y* axis. The slopes of the fitted lines are the tangent elastic modulus (see Table 3). The strain difference between the stress–strain curves at different saturation is used to quantify the wetting deformation of soil, as follows:

2 $$\varepsilon _{1}^{{{\text{sh}}}}=\varepsilon _{1}^{{_{{{S_{{\text{r1}}}}}}}} - \varepsilon _{1}^{{_{{{S_{{\text{r2}}}}}}}}={\left[ {\frac{{\left( {{\sigma _1} - {\sigma _3} - t} \right)}}{{{E_{\text{t}}}}}} \right]_{{S_{{\text{r1}}}}}} - {\left[ {\frac{{\left( {{\sigma _1} - {\sigma _3} - t} \right)}}{{{E_{\text{t}}}}}} \right]_{{S_{{\text{r2}}}}}}$$

where ${S_{{\text{r}}1}}$ and ${S_{{\text{r2}}}}$ represent different saturations of soil specimens, ${S_{{\text{r}}1}}$>${S_{{\text{r2}}}}$, ${S_{{\text{r}}1}}$ = 100%. The method is suitable for analyzing both saturated and unsaturated soils within the studied range. The results of wetting deformation (*ε*sh 1) of GRS from the unsaturated to saturated state were calculated, as shown in Figs. 12 and 13.

**Table 3:** Values of T.e parameters *E*_t_ and *t*.

Unloading path	S_*r*_ (%)	σ_3_ (kPa)	E_t_ (kPa)	t (kPa)
Constant σ_1_ unloading σ_3_	40	50	180.26	63.90
		100	523.24	128.39
		200	488.32	255.30
	80	50	63.39	63.27
		100	196.77	128.43
		200	83.09	246.50
	100	50	50.54	60.93
		100	67.70	126.00
		200	64.82	246.85
Unloading σ_1_ constant σ_3_	40	50	220.93	47.12
		100	254.23	81.76
		200	842.62	142.05
	80	50	103.07	39.24
		100	133.60	79.56
		200	664.20	128.46
	100	50	75.32	47.74
		100	114.99	89.28
		200	250.39	167.03

The wetting deformation of soil under a certain deviatoric stress σ_w_ can be calculated according to Eq. (2). It is noteworthy that the method for calculating the wetting deformation of soil using Eqs. (1) and (2) is applicable to the case where the deviatoric stress σ_w_ is not greater than the strength of the soil itself. The wetting deformation allowed in the soil during the excavation process of foundation pits is much smaller than the ultimate strain when the soil strength fails. In this context, Eq. (2) in this study has wide applicability. It can be seen from Figs. 12 and 13 that the lower the saturation of the soil sample, the greater the suction, and the greater the wetting deformation.

The relationship between the tangent elastic modulus *E*_t_ and the saturation *S*_r_ and confining pressure σ_3_ was obtained by dualistic linear regression analysis as:

3 $${E_{\text{t}}}={A_1}{P_a}{S_{\text{r}}}+{B_1}{\sigma _3}+{\beta _1}$$

where: *A*_1_ and *B*_1_ are parameters related to the saturation *S*_r_ and confining pressure σ_3_, respectively; and *P*_*a*_ is the atmospheric pressure, which has the same unit as σ_3_. The effect of introducing *P*_*a*_ is to make the parameter *A*_1_ dimensionless. The values of these parameters are shown in Table 4.

**Table 4:** Values of *A*_1_, *B*_1_, and *β*_1_.

Unloading path	A_1_	B_1_	β_1_ (kPa)
Constant σ_1_, unloading σ_3_	− 581.38	0.58	549.66
Unloading σ_1_, constant σ_3_	− 467.29	3.18	266.72

It can be seen from Table 4 that saturation has a significant influence on the tangent elastic modulus of soil under different unloading paths. When the unloading path was constant σ_1_ and unloading σ_3_, the absolute value of *A*_1_ was significantly higher. This indicates that saturation had a dominant influence on the tangent modulus under these conditions. Saturation impacts the stiffness of soil by influencing its suction, where increased saturation reduces suction and consequently decreases the tangent elastic modulus. Conversely, when the unloading path was unloading σ_1_ and constant σ_3_, the value of *B*_1_ become more prominent. This indicates that confining pressure had a stronger impact on the tangent elastic modulus under these conditions, reducing the relative significance of saturation effects.

#### Hyperbolic model

The hyperbolic model can be used to represent the stress–strain relationship of soil with high reliability. Therefore, in this paper, the hyperbolic model was used to represent the stress–strain relationship of GRS under the unloading paths as follows:

4 $${\sigma _1} - {\sigma _3}=\frac{{{\varepsilon _1}}}{{a+b{\varepsilon _1}}}+{\left( {{\sigma _1} - {\sigma _3}} \right)_0}$$

Rearranging Eq. (4) gives:

5 $$\frac{{{\varepsilon _1}}}{{\left( {{\sigma _1} - {\sigma _3}} \right) - {{\left( {{\sigma _1} - {\sigma _3}} \right)}_0}}}=a+b{\varepsilon _1}$$

For Eqs. (4) and (5), the limits were obtained as:

6 $$\mathop {\lim }\limits_{{{\varepsilon _1} \to \infty }} \left[ {\frac{{{\varepsilon _1}}}{{a+b{\varepsilon _1}}}+{{\left( {{\sigma _1} - {\sigma _3}} \right)}_0}} \right]=\frac{1}{b}+{\left( {{\sigma _1} - {\sigma _3}} \right)_0}={\left( {{\sigma _1} - {\sigma _3}} \right)_{\lim }}$$

7 $$\mathop {\lim }\limits_{{\begin{array}{*{20}{c}} {{\varepsilon _1} \to 0} \\ {\left( {{\sigma _1} - {\sigma _3}} \right) \to {{\left( {{\sigma _1} - {\sigma _3}} \right)}_0}} \end{array}}} \left[ {\frac{{{\varepsilon _1}}}{{\left( {{\sigma _1} - {\sigma _3}} \right) - {{\left( {{\sigma _1} - {\sigma _3}} \right)}_0}}}} \right]=\mathop {\lim }\limits_{{\begin{array}{*{20}{c}} {{\varepsilon _1} \to 0} \\ {\left( {{\sigma _1} - {\sigma _3}} \right) \to {{\left( {{\sigma _1} - {\sigma _3}} \right)}_0}} \end{array}}} \frac{{d{\varepsilon _1}}}{{d\left[ {\left( {{\sigma _1} - {\sigma _3}} \right) - {{\left( {{\sigma _1} - {\sigma _3}} \right)}_0}} \right]}}=\frac{1}{{{E_{\text{i}}}}}=a$$

where: (σ_1_ − σ_3_)_0_ is the initial deviatoric stress of the soil specimen after *K*_0_-consolidation was completed; *a* is the reciprocal of the initial tangent modulus *E*_i_; and *b* is the reciprocal of the deviatoric stress [(σ_1_ − σ_3_)_lim_ − (σ_1_ − σ_3_)_0_].

The relationship curves of *ε*_1_ / [(σ_1_ − σ_3_) − (σ_1_ − σ_3_)_0_] and *ε*_1_ under different confining pressures for different unloading paths were plotted to obtain the parameters *a* and *b*, where the intercept with the *y* axis is *a* and the slope is *b*. These parameters are shown in Table 5.

**Table 5:** Hyperbolic model parameters of soil specimens under different unloading paths.

*S*_r_ (%)	σ_3_ (kPa)	Constant σ_1_, unloading σ_3_						Unloading σ_1_, constant σ_3_					
		(σ_1_ − σ_3_)_f_ (kPa)	(σ_1_ − σ_3_)_lim_ (kPa)	*R*_f_		*a* (10^− 3^ kPa^− 1^)	*b* (10^− 3^ kPa^− 1^)	(σ_1_ − σ_3_)_f_ (kPa)	(σ_1_ − σ_3_)_lim_ (kPa)	*R*_f_		*a* (10^− 3^ kPa^− 1^)	*b* (10^− 3^ kPa^− 1^)
				Tested value	Average					Tested value	Average		
40	50	–	116.31	–	0.956	2.38	17.76	−50	−54.42	0.919	0.921	1.40	−8.74
	100	–	234.68	–		1.32	8.72	−92	−106.24	0.866		0.90	−4.42
	200	410	428.68	0.956		0.96	5.30	−158	−161.61	0.978		0.40	−2.49
80	50	99	101.31	0.977	0.976	7.72	24.21	−46	−51.11	0.900	0.920	2.31	−9.00
	100	–	176.72			3.98	17.63	−70	−74.55	0.939		1.36	−5.14
	200	315	323.26	0.974		2.55	12.01	−148	−127.65	—		0.63	−2.72
100	50	86	88.92	0.967	0.942	12.52	34.58	−45	−47.30	0.951	0.925	4.46	−9.22
	100	142	162.59	0.873		9.07	23.48	−66	−73.42	0.899		2.15	−5.17
	200	292	296.24	0.986		5.58	17.78	−139	−126.30	—		0.85	−2.73

Note: *R*_f_ is the failure stress ratio, *R*_f_ = (σ_1_ − σ_3_)_f_ / (σ_1_ − σ_3_)_lim_.

Rearranging Eq. (4) gives:

8 $${\varepsilon _1}=\frac{a}{{\frac{1}{{\left( {{\sigma _1} - {\sigma _3}} \right) - {{\left( {{\sigma _1} - {\sigma _3}} \right)}_0}}} - b}}$$

The difference between the two stress–strain curves under the same unloading path and same confining pressure is the wetting deformation, defined as:

9 $$\varepsilon _{1}^{{{S_{{\text{r1}}}}}} - \varepsilon _{1}^{{{S_{{\text{r2}}}}}}=\frac{{{a_{{S_{{\text{r1}}}}}}}}{{\frac{1}{{{{\left[ {\left( {{\sigma _1} - {\sigma _3}} \right) - {{\left( {{\sigma _1} - {\sigma _3}} \right)}_0}} \right]}_{{S_{{\text{r1}}}}}}}} - {b_{{S_{{\text{r1}}}}}}}} - \frac{{{a_{{S_{{\text{r2}}}}}}}}{{\frac{1}{{{{\left[ {\left( {{\sigma _1} - {\sigma _3}} \right) - {{\left( {{\sigma _1} - {\sigma _3}} \right)}_0}} \right]}_{{S_{{\text{r2}}}}}}}} - {b_{{S_{{\text{r2}}}}}}}}$$

where ${S_{{\text{r}}1}}$ and ${S_{{\text{r2}}}}$ represent different unsaturations of soil specimens, ${S_{{\text{r}}1}}$>${S_{{\text{r2}}}}$, ${S_{{\text{r}}1}}$ = 100%.

**Fig. 14:**
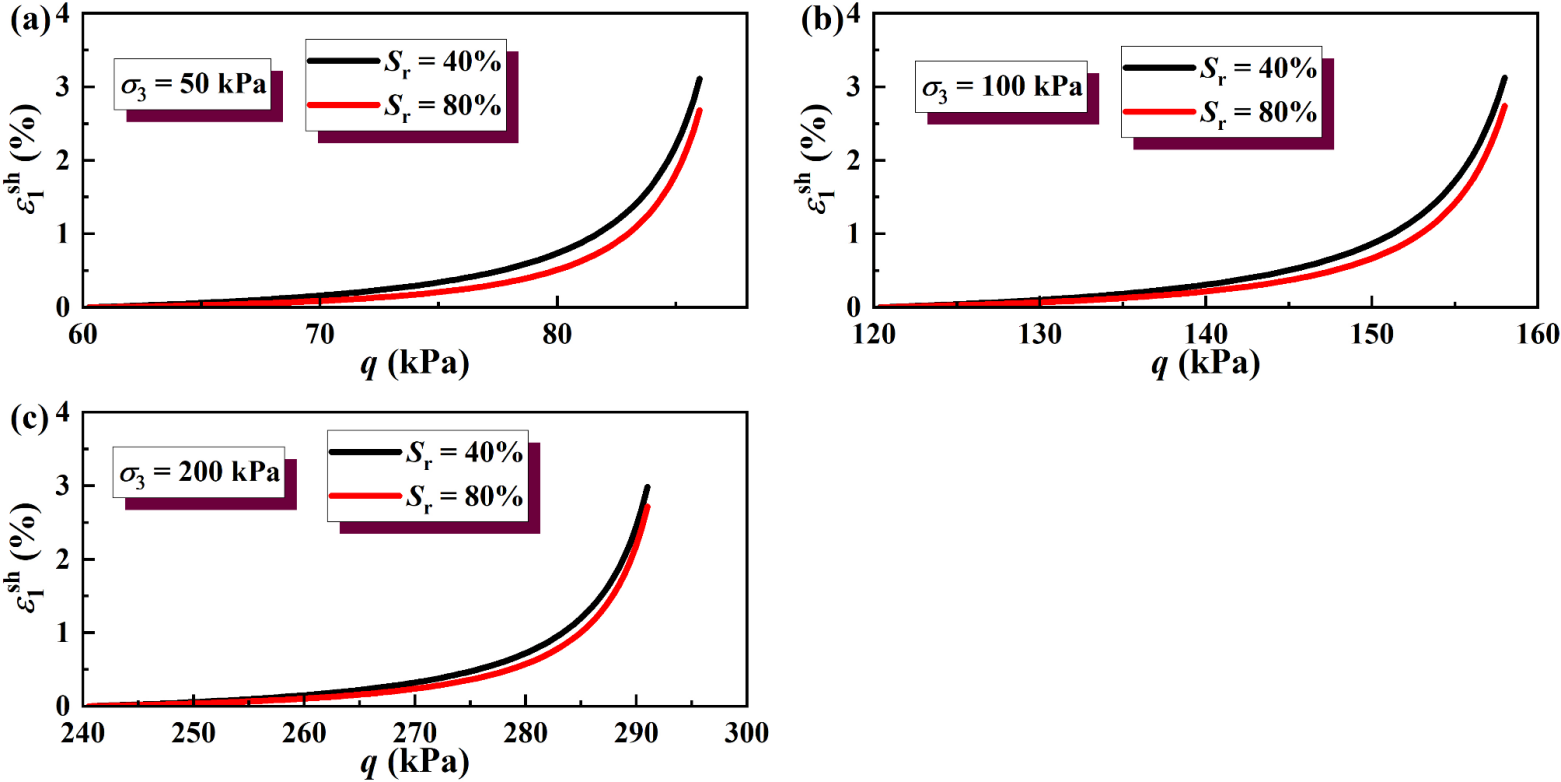
Wetting deformation under unloading σ_3_ calculated by the hyperbolic model: (**a**) σ_3_ = 50 kPa; (**b**) σσ_3_ = 100 kPa; (**c**) σ_3_ = 200 kPa.

**Fig. 15:**
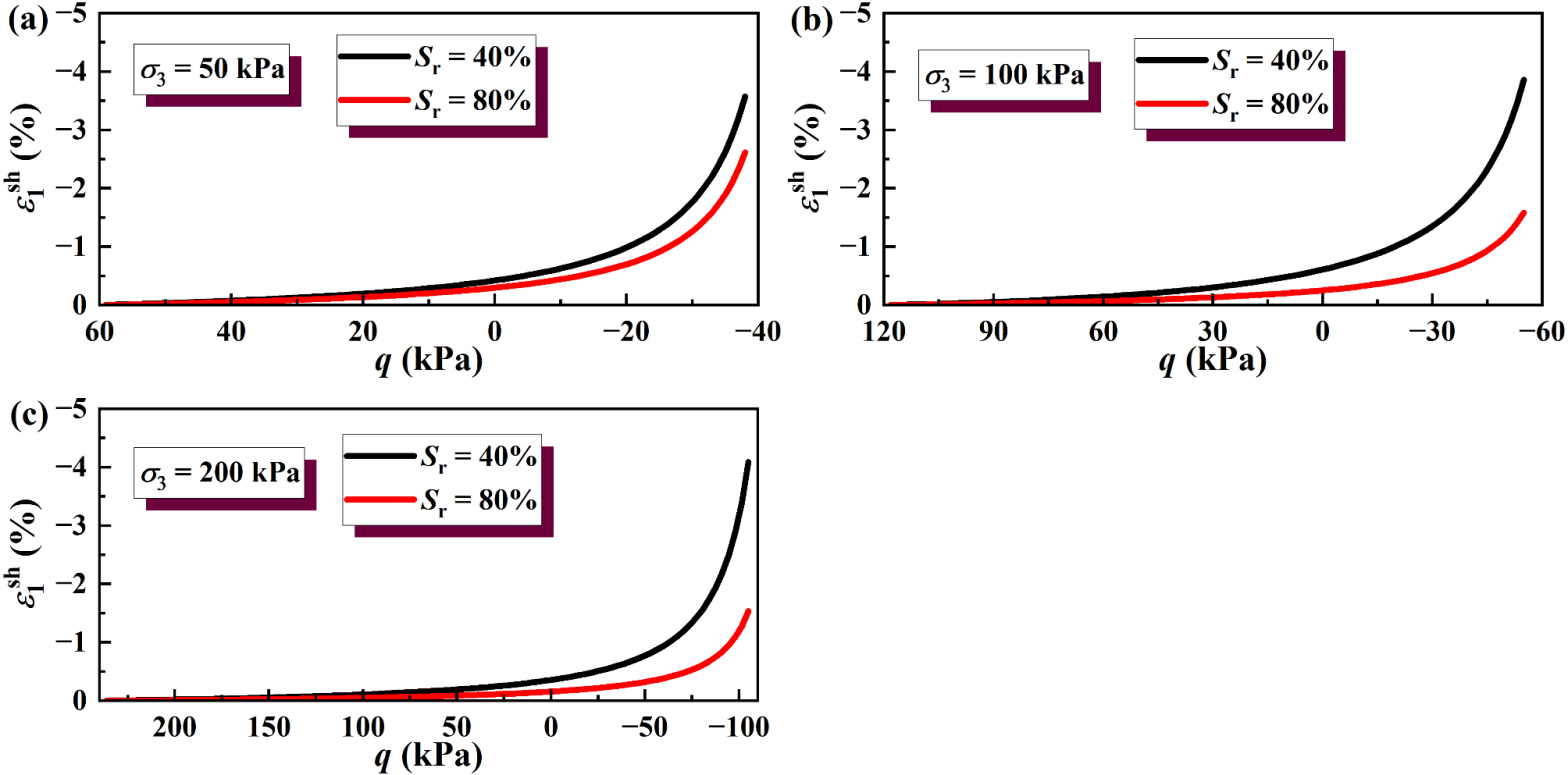
Wetting deformation under unloading σ_1_ calculated by the hyperbolic model: (**a**) σ_3_ = 50 kPa; (**b**) σ_3_ = 100 kPa; (**c**) σ_3_ = 200 kPa.

The results of the wetting deformation (*ε*sh 1) of GRS from the unsaturation to saturation state were calculated, and the results are shown in Figs. 14 and 15. The results of soil wetting deformation calculated by the hyperbolic model were generally consistent with those calculated by the simplified linear model. Both show that the wetting deformation of low-saturation soil wetting to saturation was greater than that of high-saturation soil wetting to saturation.

**Fig. 16:**
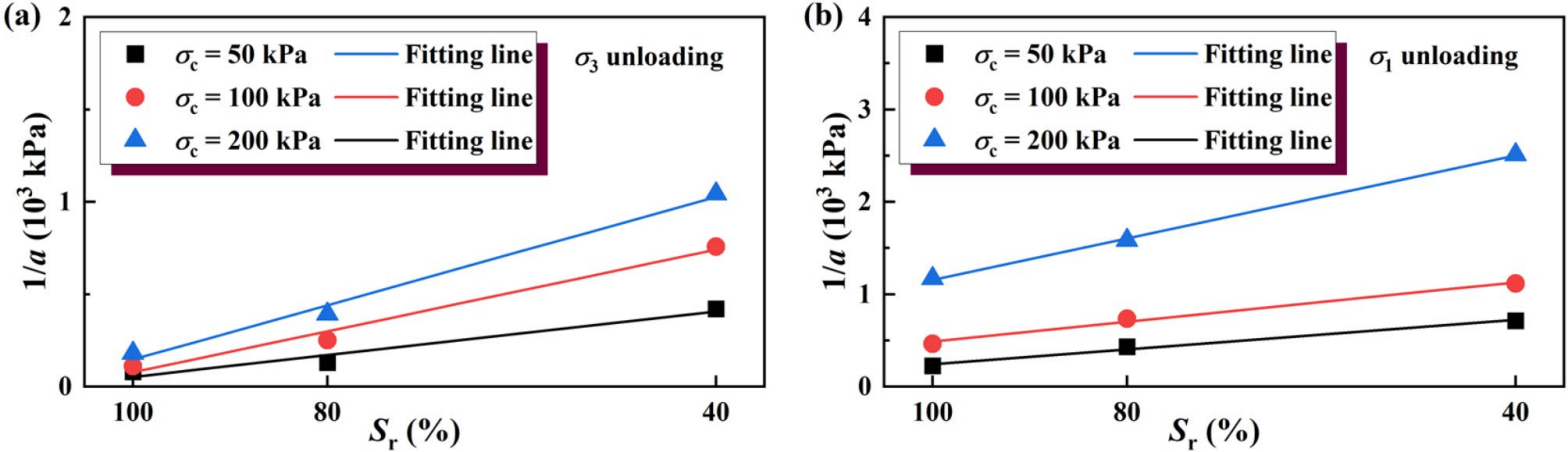
Relationship between 1/*a* (initial tangent modulus) and *S*_r_: (**a**) unloading σ_3_; (**b**) unloading σ_3_.

Figure 16 shows a linear relationship between the initial tangent modulus *E*_i_ (equal to 1/*a*) and the saturation *S*_r_ under different confining pressure. As *S*_r_ decreases, the matric suction within the soil increases significantly, enhancing interparticle forces and improving the deformation stiffness. This effect is particularly evident in unsaturated soils, where lower saturation conditions lead to higher resistance against deformation. The deformation stiffness in the early shear stage, represented by the initial tangent modulus *E*_i_, is a critical indicator of soil behavior under loading. Figure 16 shows that higher confining pressures and lower saturation levels contribute to increased *E*_i_, indicating greater resistance to deformation during the onset of shear. This trend highlights the importance of considering both saturation and confining pressure in predicting soil stiffness during excavation or loading scenarios. Conversely, at lower confining pressures, the degree of consolidation is reduced, leading to looser soil packing and lower deformation stiffness in the initial shear phase. This condition may benefit foundation pit excavation by reducing the energy required for soil removal. However, the reduced stiffness can lead to higher deformation risks, necessitating careful consideration of stability measures in engineering applications.

The binary linear regression analysis was applied to investigate how saturation and confining pressure jointly affect the parameter a:

10 $$a={A_2}{P_{\text{a}}}{S_{\text{r}}}+{B_2}{\sigma _{3{\text{c}}}}+{\beta _2}$$

where: *A*_2_ and *B*_2_ are parameters related to saturation *S*_r_ and confining pressure σ_3_, respectively; and *P*_*a*_ is the atmospheric pressure, which has the same unit as σ_3_. The effect of introducing *P*_*a*_ is to make the parameter *A*_2_ dimensionless. The values of these parameters are shown in Table 6.

**Table 6:** Values of *A*_2_, *B*_2_, and *β*_2_.

Unloading paths	A_2_ (kPa^− 2^)	B_2_ (kPa^− 2^)	β_2_ (kPa^− 1^)
Constant σ_1_, unloading σ_3_	0.01161	−2.90936 × 10^− 5^	4.629 × 10^− 5^
Unloading σ_1_, constant σ_3_	0.00246	−1.31742 × 10^− 5^	1.34 × 10^− 3^

The tangent elastic modulus *E*_t_ can be expressed as:

11 $${E_{\text{t}}}={E_{\text{i}}}{\left( {1 - {R_{\text{f}}}S} \right)^2}$$

where

12 $$S=\frac{{{\sigma _1} - {\sigma _3}}}{{{{\left( {{\sigma _1} - {\sigma _3}} \right)}_{\text{f}}}}}$$

The Mohr–Coulomb strength relationship is:

13 $${\left( {{\sigma _1} - {\sigma _3}} \right)_{\text{f}}}=\frac{{2c\cos \varphi +2{\sigma _{3{\text{c}}}}\sin \varphi }}{{1 - \sin \varphi }}$$

The tangent elastic modulus can be expressed as follows using Eqs. (11), (12), and (13):

14 $${E_{\text{t}}}={E_{\text{i}}}{\left[ {1 - \frac{{{R_{\text{f}}}\left( {1 - \sin \varphi } \right)\left( {{\sigma _1} - {\sigma _3}} \right)}}{{2c\cos \varphi +2{\sigma _{3{\text{c}}}}\sin \varphi }}} \right]^2}$$

where *c* is the cohesion of the soil and *ϕ* is its angle of internal friction.

The regression parameters *A*_2_ and *B*_2_ (Table 6) reveal that both a decrease in saturation and confining pressure lead to a reduction in *a*. Additionally, the larger value of *A*_2_ compared to *B*_2_ indicates that saturation has a more pronounced influence on *a*.

It can be seen from Table 6 that, similar to the calculation results of the simplified linear model, the hyperbolic model also shows a phenomenon whereby the saturation of soil specimens and the confining pressure of the tests have different effects on the tangent elastic modulus under different unloading paths. However, in contrast to the calculation results of the simplified linear model, the results of the hyperbolic model show that when the unloading path was constant σ_1_ and unloading σ_3_, the influences of the soil saturation and the test confining pressure on the tangential elastic model were more prominent.

**Fig. 17:**
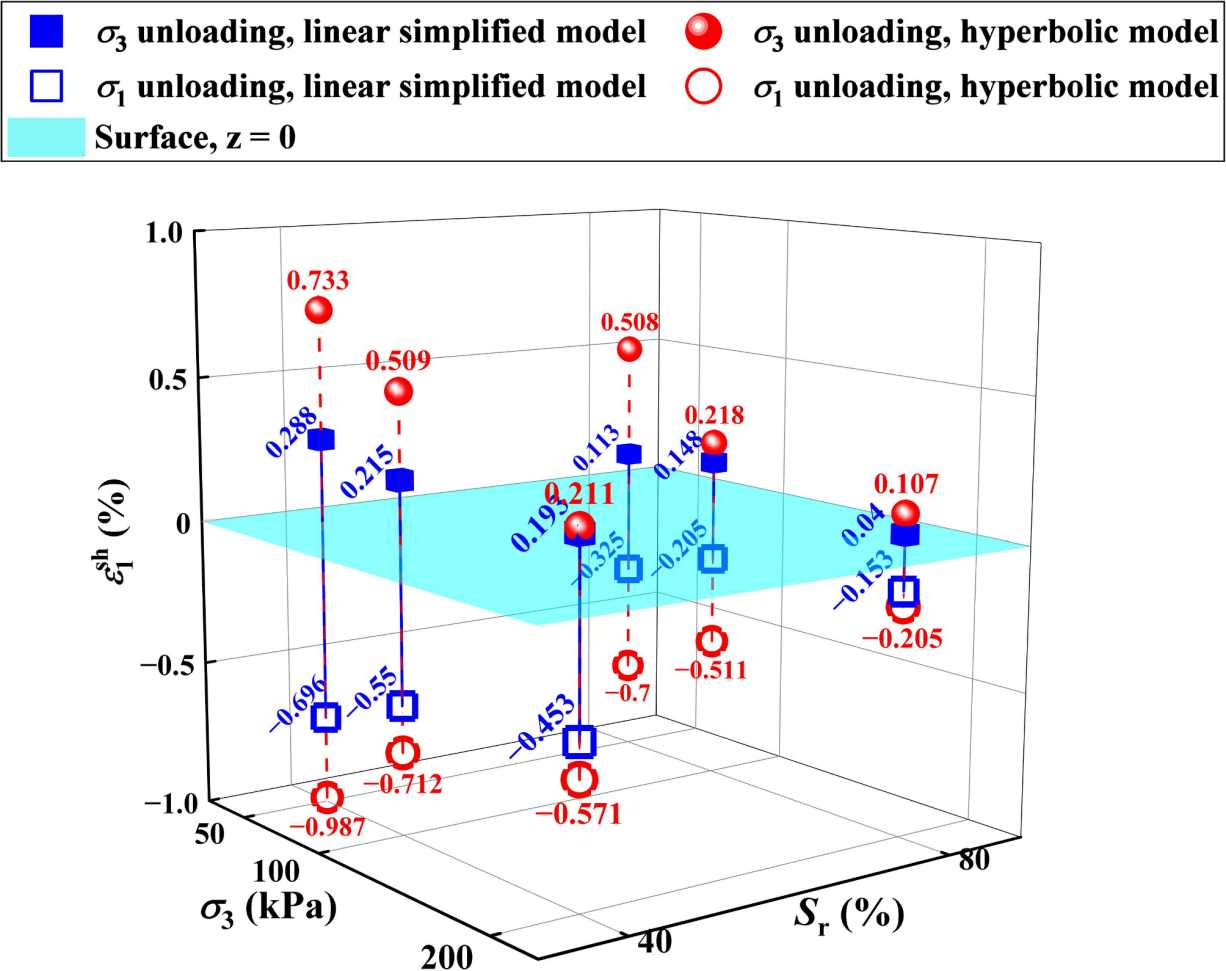
Wetting deformations of soil calculated by two different models. Note: the wetting deformation was positive in compression and negative in tension. The wetting deformation here refers to the wetting deformation at a certain deviatoric stress *q* = σ_1_ − σ_3_. When σ_3_ is unloading, *q* = *q*_0_ + 20 kPa; when σ_1_ is unloading, *q* = − 20 kPa.

#### Comparison of calculation results of different models

To compare the difference in the wetting deformations of GRS calculated by two different analysis methods more intuitively, the data were plotted on 3D axes, as shown in Fig. 17. It can be seen that the results of both calculation models indicate that the wetting deformation of the soil increases with decreasing saturation under the same confining pressure; conversely, the wetting deformation of the soil at a given saturation state decreases with increasing confining pressure. As previously discussed, higher confining pressure mitigates the wetting deformation by reducing the detrimental effects of water on the iron-oxide cement in GRS.

Although the simplified linear model provides a straightforward method for estimating wetting deformation, it does not fully capture the observed nonlinear trends in stress-strain behavior, as shown in Fig. 11. The deviation from linearity becomes more pronounced at higher saturation levels, where the wetting deformation accelerates due to progressive weakening of interparticle bonding.

In contrast, the hyperbolic model accounts for this nonlinear deformation behavior, making it more suitable for wetting deformation predictions under unloading conditions. The fitting results in Fig. 17 indicate that the hyperbolic model achieves a higher accuracy compared to the simplified linear model, confirming its superiority in practical applications. These findings highlight the necessity of using a nonlinear approach, such as the hyperbolic model, for predicting foundation pit deformation under coupled unloading-wetting conditions.

It also can be seen from Fig. 17 that under the same confining pressure and saturation to saturation state, the wetting deformations under different unloading paths were different, showing that the tensile was greater than the compression. This also confirms the spatial variability and anisotropy of natural GRS as structural soil. In addition, with increasing consolidation confining pressure, the anisotropy of GRS will be reduced, this phenomenon was also observed by Liu et al.^32^.

The anisotropic behavior of wetting deformation is influenced not only by the initial stress state but also by the inherent fabric anisotropy of GRS, stress path effects, and suction-related mechanisms. The in-situ weathering process results in a directional soil structure, which affects how deformation develops under different unloading conditions.

Additionally, stress redistribution during unloading plays a crucial role in deformation anisotropy. Under vertical unloading, soil particles remain more constrained due to the overburden effect, whereas horizontal unloading allows for greater particle rearrangement and deformation. Furthermore, the loss of matric suction during wetting alters the interparticle forces differently in different directions, further contributing to anisotropic deformation behavior.

The experimental results show that wetting-induced elongation deformation is significantly greater than compressive deformation, and this anisotropic effect diminishes with increasing confining pressure. This reduction is associated with the damaged cementation and the changed fissures. These findings highlight the need to consider stress-path-dependent anisotropy in wetting deformation analysis, particularly for excavation stability assessments.

However, it is important to note that the choice of model should consider specific project conditions, such as soil type and stress history, as these factors may influence the model’s accuracy. Future research could explore the performance of alternative models under similar conditions to further validate the findings.

## Discussion

**Fig. 18:**
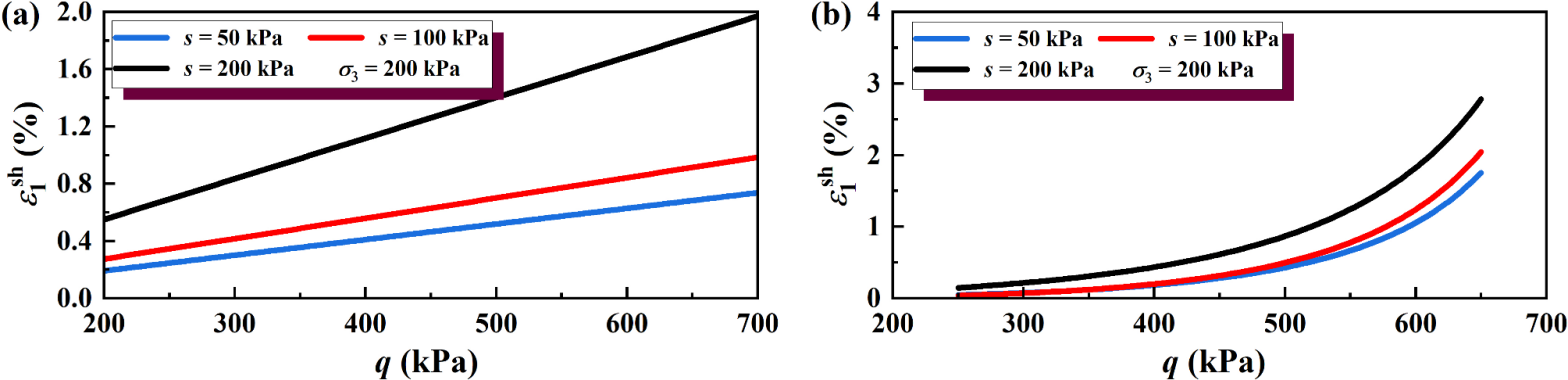
The wetting deformation of loess calculated by different models (where, *s* is suction of soil): (**a**) simplified linear model; (**b**) hyperbolic mode^28^.

In this work, the simplified linear model and the hyperbolic model were used to analyze the deformation behavior of GRS under the coupling of unloading and wetting. The same research method was used to study the wetting deformation characteristics of loess (from Gansu, China) by Guan et al.^28^, and the test results as shown in Fig. 18. The calculation results show that the wetting deformation of loess increases with the increase of deviatoric stress. Under the same deviatoric stress, the wetting deformation of loess with a lower suction (meaning higher saturation of the soil) was significantly smaller than that with higher suction. The calculation results of the simplified linear model and the hyperbolic model were consistent with the wetting-deformation characteristics of the soil. The results of Guan et al.^28^ are in substantial agreement with those of this work.

It is worth noting that the two studies employed different stress paths. Guan et al.’s^28^ research adopted a loading path, while this study used an unloading path. Whether the computational model developed under the loading path is applicable to the unloading path remains an unanswered question in previous research.

Furthermore, Guan et al.’s^28^ study employed isotropic consolidation, whereas this study used *K*_0_-consolidation (i.e., anisotropic consolidation). The model in this study builds upon Guan et al.’s^28^ framework by accounting for the presence of initial deviatoric stress, which arises from anisotropic consolidation. The introduction reviewed the influence of consolidation methods on the mechanical behavior of natural GRS, underscoring the importance of employing *K*_0_-consolidation in laboratory tests.

Therefore, the significance of the fitted model in this study is twofold. First, it validates that the theoretical framework of the wetting deformation computational model under the loading path is applicable to the wetting deformation calculation of GRS under the unloading path, which is very important. Second, the model incorporates the presence of initial deviatoric stress into the original framework, providing a wetting deformation computational model suitable for *K*_0_-consolidation. This advancement offers practical guidance for engineering applications.

However, it is worth noting that the wetting deformation of loess was greater than that of GRS under the same confining pressure. The possible reason was that the mechanism of wetting deformation of Gansu loess and Shenzhen GRS was different. The basic unit shape of Gansu loess is granular, and the granular units are connected by contact-cementation. With the intrusion of water, the strength of the soil particle connection point is weakened. Then, the surrounding soil particles fall into the hole, causing the wetting deformation of loess, this was also confirmed in research of Chen et al.^9^. Unlike Gansu loess, the basic unit forms of GRS are granular and flaky. The units are connected by free iron oxide gel, and a large amount of free iron oxide is in the colloidal state. Then, with the intrusion of water, the dense colloidal iron oxide is diluted, resulting in the weakening of the soil’s resistance to deformation and the wetting deformation of the soil. For undisturbed soils, wetting deformation occurs through different mechanisms. In loess, the rapid collapse of voids due to the weakening of cementation bonds leads to sudden settlement and erosion, which can even progress to large-scale collapse under sufficient infiltration^35^. In contrast, GRS exhibits a slower but cumulative deformation process as colloidal iron oxide bonds are diluted. Although GRS shows a lower immediate risk of collapse, prolonged exposure to water can result in significant cumulative settlement, highlighting its vulnerability in long-term scenarios. So, different wetting deformation mechanisms of soil lead to stronger wetting deformation behavior of Gansu loess, and its anti-deformation ability is more strongly affected by water.

Furthermore, the excavation of foundation pits introduces significant anisotropic stress redistribution, which amplifies wetting deformation near excavation boundaries. Rainfall during excavation can further reduce matric suction, accelerating the weakening of particle bonds and leading to localized deformation. In contrast, pre-excavation rainfall or groundwater infiltration typically results in more uniform wetting deformation. These differences underscore the importance of considering stress-path effects in the analysis of wetting deformation during foundation pit design.

In addition, wetting deformation varies significantly under different conditions. For instance, during rainfall or surface infiltration, the isotropic stress state primarily leads to uniform settlement near the surface, gradually propagating deeper. Groundwater intrusion causes layered deformation as the water level rises, with isotropic stress redistribution across the affected layers. During excavation, however, the anisotropic stress redistribution caused by unloading amplifies deformation near excavation boundaries, particularly under concentrated stress zones. These differences highlight the critical role of stress-path and water entry conditions in wetting deformation analysis.

In addition, it is widely accepted that the stress release of the soil element occurs during the excavation of the foundation pit. This work studied the wetting-deformation behavior of the soil under the unloading stress paths. It is found that the wetting-deformation model under the loading stress path given in previous studies is also applicable to the prediction of the wetting deformation of the soil under the unloading stress path. However, this finding is rarely mentioned in previous studies. The findings of this study have practical implications for controlling wetting deformation in foundation pit excavations. By understanding the coupled effects of unloading and wetting, engineers can adopt targeted measures to mitigate deformation risks. For instance, the implementation of effective drainage systems can reduce saturation increases during excavation, and the adjustment of support structure stiffness based on the predicted deformation can enhance stability. Moreover, soil improvement techniques such as grouting or chemical stabilization can be considered for highly sensitive soils like GRS. These measures, combined with real-time monitoring, provide a comprehensive approach to controlling wetting deformation in excavation projects.

It should be noted that this work has examined wetting-deformation characteristics of GRS only via the double-line test. While the double-line method was employed in this study to isolate and quantify the coupled effects of unloading and wetting deformation, it is acknowledged that the single-line method may better represent real engineering conditions. Future studies will integrate single-line method tests to complement these findings and provide a more comprehensive understanding of wetting deformation behavior. GRS exhibits regional characteristics, and the wetting deformation behavior during excavation may vary across different regions. Therefore, further research is required to investigate the wetting deformation characteristics of GRS in other regions.

## Conclusion

Based on stress changes in the soil during the excavation of a foundation pit, this work examined the wetting-deformation characteristics of GRS under two unloading paths using the double-line method. The simplified linear model and hyperbolic model were used to calculate the wetting deformation of GRS. The main conclusions that were obtained are as follows.

(i) The stress–strain curves of natural GRS under two unloading paths were found to be of the strain-hardening type, distinguishing them from typical loading-induced responses. Under a given confining pressure, with increasing saturation, the maximum deviatoric stress of the soil decreased significantly, particularly at lower confinement levels. The impact of confining pressure on soil strength enhancement was more pronounced under lower saturation levels, indicating a stress-dependent weakening effect. These findings provide new insights into the coupled behavior of unloading and wetting deformation in GRS.
(ii) The results of the soil wetting deformation calculations of both models showed that the deformation of GRS increases with decreasing soil saturation (increasing soil suction), and the deformation decreases with increasing confining pressure.
(iii) The wetting deformation of GRS calculated by the hyperbolic model was greater than that calculated by the simplified linear model. To ensure the safety of foundation-pit excavations, the hyperbolic model should be used to calculate the deformation under the coupled effects of unloading and wetting at the design stage.
(iv) GRS has wetting deformation anisotropy; in the process of wetting, its tensile deformation potential was found to be greater than the compression deformation. The anisotropy effect of GRS was weakened with increasing confining pressure. The wetting deformation values calculated by the hyperbolic model were found to be safer in actual engineering design.

This work studied the wetting-deformation behavior of the soil under the unloading stress paths. However, it is important to acknowledge that these results were obtained from controlled laboratory tests and may not comprehensively represent all aspects of saturated and unsaturated soil behavior. To further validate the applicability of these models, additional experiments under different stress paths and saturation conditions will be conducted.

## Data Availability

The datasets used and analysed during the current study available from the corresponding author on reasonable request.
